# Naturally derived hydrogel with antioxidant, angiogenesis and photothermal effect to accelerate infected diabetic wound healing and reduce scar formation

**DOI:** 10.7150/thno.117179

**Published:** 2025-09-08

**Authors:** Mengyu Yang, Pengyuan Liu, Pei Cheng, Chenghao Li, Jingmei Liu, Haifeng Sun, Fangxia Guan, Minghao Yao

**Affiliations:** 1 School of Life Science, Zhengzhou University, 100 Science Road, Zhengzhou 450001, P. R. China.; 2 Department of Colorectal and Anal Surgery, The First Affiliated Hospital of Zhengzhou University, Zhengzhou 450001, P. R. China.

**Keywords:** Diabetic wound healing, Photothermal antibacterial, Multifunctional hydrogel, ROS scavenging hydrogel, Less scar repair

## Abstract

**Rationale:** Diabetic wound treatment remains a central issue in global healthcare due to the unitary nature of clinical dressings, which lack systemic multifunctionality in terms of tissue adhesion, shape adaptation, hemostasis, antioxidant, anti-inflammatory and antimicrobial abilities and promotion of tissue repair.

**Methods:** A hydrogel dressing loaded with active molecules of traditional Chinese medicine has been developed for the treatment of infected diabetic wounds. The naturally derived multifunctional hydrogel dressing (abbreviated as CTP) composed of Carboxymethyl chitosan (CMCS), 3,4,5-Trihydroxybenzaldehyde (THBA), and Phlorizin (PHL) was fabricated by a simple mixing process avoiding the use of any additional cross-linking agents or functional modifications.

**Results:** The developed hydrogel demonstrated adhesiveness, shape adaptability, swelling, photothermal responsiveness, and other desirable biological functions, including hemostatic behavior and antibacterial, antioxidant and proangiogenic activities. In a diabetic wound model, compared with Hydrosorb Gel (a commercial hydrogel wound dressing), the CTP hydrogel in combination with NIR treatment accelerated diabetic wound healing by effectively preventing infection, decreasing inflammatory response, promoting re-epithelialization, enhancing both regeneration of skin attachments (e.g., hair follicles) and deposition of collagen. It also reduced scar formation and improved the overall healing process.

**Conclusions:** As a functional wound dressing, CTP hydrogel shows great promise in diabetic wound repair.

## Introduction

Diabetes is a metabolic disease with a high global prevalence, characterized by hyperglycemia due to defective or dysfunctional insulin secretion 1. Patients with diabetes are often confronted with the formation of chronic wounds or ulcers, which are difficult to heal and become a major challenge in clinical management 2. In diabetic patients, a hyperglycemic environment can negatively affect the wound healing process 3. Firstly, diabetes leads to pathologic changes in blood composition that may trigger persistent bleeding and increase the risk of bacterial infection 4. Secondly, the persistence of abnormally functioning macrophages and neutrophils at diabetic wound sites leads to exacerbation and prolongation of the inflammatory phase 5. At the same time, inflammatory cells recruited at the wound site produce high levels of reactive oxygen species (ROS), which in turn induces the occurrence of oxidative stress 6, 7. In addition, a high-glucose environment affects fibroblast migration and neovascularization, reducing blood supply and nutrient delivery to the wound site 8. Finally, the abnormal tissue remodeling process can lead to excessive scar proliferation, which impairs functional recovery and aesthetics of the wound 9.

During wound healing, bacterial infection has been shown to be one of the critical factors affecting the healing process 10. Diabetic wounds are more susceptible to bacterial infection due to impaired immune function 6. Photothermal therapy (PTT) not only avoids the problem of drug resistance due to antibiotic abuse, but also has target selectivity, temporal and spatial controllability, and minimally invasiveness 11-14. It has been shown that polyphenols have favorable effects in photothermal effect, antimicrobial, antioxidant, and immunomodulation 15. Therefore, it is expected that the photothermal property of polyphenols can be introduced into wound dressings to enhance the therapeutic effect in diabetic wound healing. In addition, inhibition of scar formation is also an important aspect that must be considered in wound dressing application. Excessive skin scarring not only weakens the function of skin tissues, but also may pose a heavy economic burden 16. Excessive production of ROS during wound healing is one of the key factors leading to scar formation 17. In diabetic patients, ROS significantly alter the biology of the extracellular matrix (ECM) through glycosylation and oxidative reactions, thereby promoting scar formation 18. In order to minimize scarring, excessive ROS need to be effectively prevented from triggering oxidative stress and adverse inflammatory responses 19.

In the field of wound healing, novel hydrogels developed by skillfully combining active molecules of traditional Chinese medicine with functional polymers have become a hot research topic 20. For example, resveratrol has been widely studied for its excellent antioxidant, anti-inflammatory and pro-angiogenic properties 21. However, resveratrol hydrogel systems still face challenges in bioavailability enhancement and multi-bond network construction. Curcumin hydrogel, on the other hand, have achieved multifaceted modulation of the wound microenvironment by combining natural polysaccharides such as heparin and hyaluronic acid 22. Nevertheless, the water solubility and stability issues of curcumin limit its potential in clinical applications. Gallic acid hydrogel significantly accelerated the healing of infected diabetic wounds through its photothermal effect and antioxidant capacity 23. However, this system still needs to be further explored in terms of antimicrobial mechanism and long-term biocompatibility. In contrast to these studies, Phlorizin (PHL) possesses a variety of biological activities, such as antioxidant, anti-inflammatory, antibacterial, and blood glucose regulation 24. Its excellent antioxidant capacity can effectively scavenge ROS at the wound site and also further promote the wound healing process by inhibiting excessive inflammatory responses 25. In addition, PHL can also regulate blood glucose levels by inhibiting sodium-glucose cotransporters (SGLT) 26. However, despite these excellent pharmacological properties, the low solubility of PHL causes it to exhibit low bioavailability under physiological conditions, which in turn limits its clinical application 27. Therefore, enhancing the bioavailability of PHL has become a key prerequisite for its role in promoting wound healing.

**Figure 1:**
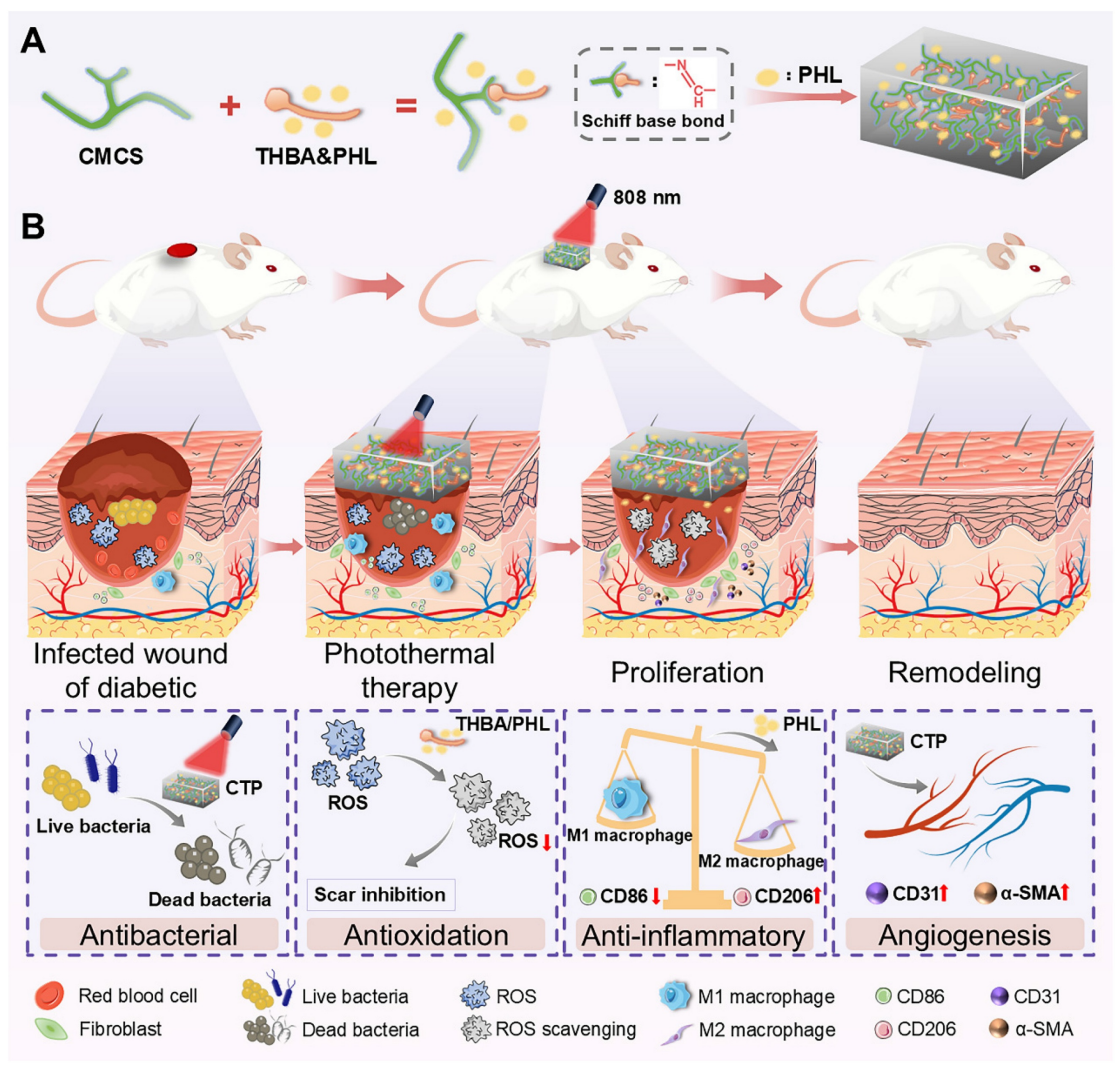
(A) Preparation of hydrogel and its components. (B) Illustration of the biological functions (antibacterial, antioxidation, anti-inflammatory, and angiogenesis effects) of the CTP enabling the effective treatment of infected diabetic wounds.

Based on the above analysis, this study designed a functional dressing for the comprehensive treatment of infected diabetic wounds. This multifunctional hydrogel (abbreviation CTP) has multiple functions such as adhesion, antioxidant, antibacterial activity, hemostatic property, promotion of cell migration and angiogenesis. As shown in Figure 1A, the skeleton of CTP hydrogel was formed by the Schiff base reaction of carboxymethyl chitosan (CMCS) and 3,4,5-Trihydroxybenzaldehyde (THBA), and PHL was further introduced. Among them, the introduction of PHL which not only enhanced the bioactivity of the hydrogel, but also broadened its application prospects in the biomedical field. The results of experiments *in vivo* further confirmed the remarkable therapeutic efficacy of the hydrogel in the treatment of infected diabetic wounds, as expressed in the reduction of inflammatory response, promotion of epithelial regeneration and angiogenesis, as well as the ability to effectively inhibit the formation of scars (Figure 1B). Thus, this multifunctional hydrogel integrates multiple desirable functions of a wound dressing through a simple method, and is expected to be an ideal choice for the treatment of infected diabetic wounds.

## Experimental Section

### Materials

Carboxymethyl chitosan (CMCS, Mw = 150-800 kDa) was purchased from Yuanye Bio-Technology Co., Ltd. (Shanghai, China). 3,4,5-Trihydroxybenzaldehyde (THBA) and Phlorizin (PHL) were obtained from Aladdin Biochemical Technology Co., Ltd. (Shanghai, China). Hydrosorb Gel was purchased from Paul Hartmann AG (Germany). Hydrogen peroxide 30% (H_2_O_2_, AR) was purchased from Kermel Chemical Reagent Co., Ltd. (Tianjin, China). All other reagents were used without further purification. Live/dead fluorescence kit (Calcein-AM/PI) was purchased from Solarbio Science & Technology Co., Ltd (Beijing, China).

### Fabrication of CTP hydrogel

CMCS powder was placed in PBS and fully dissolved at 60 °C to obtain 10% (wt/vol) CMCS solution. THBA powder was placed in PBS, dispersed by sonication and then dissolved at 45 °C to obtain 0.2% (wt/vol), 0.4% (wt/vol) and 0.6% (wt/vol) THBA. THBA and PHL powders were weighed and placed in PBS, and then dissolved by the sonication-heating cycle method. Specifically, the materials were fully sonicated and then dissolved at 45 °C. If residual components remained, they were re-sonicated and then heated, and so on, until they were completely dissolved to obtain 0.6% (wt/vol) THBA&PHL solution.

CMCS/THBA hydrogels (abbreviated as CT) were synthesized by a one-pot method by mixing equal volumes of 0.2% (wt/vol), 0.4% (wt/vol), and 0.6% (wt/vol) THBA solutions and 10% (wt/vol) CMCS solution. The final concentrations of THBA were 1 mg/mL, 2 mg/mL, and 3 mg/mL, respectively, which were correspondingly named CT_0.1_, CT_0.2_, and CT_0.3_. 0.6% (wt/vol) THBA&PHL solution and 10% (wt/vol) CMCS solution were mixed homogeneously in equal volumes to synthesize the CMCS/THBA/PHL hydrogel by the one-pot method (abbreviated as CTP), in which the final concentration of THBA was 3 mg/mL, named CT_0.3_P.

### FTIR, SEM and swelling tests of CTP hydrogel

The CMCS, THBA, PHL, CT_0.3_ hydrogel and CT_0.3_P hydrogel were prepared for Fourier transform infrared (FTIR) spectroscopy in the range of 4000 - 400 cm^-1^. Scanning electron microscope (SEM) and swelling tests are in accordance with our previous methods 28.

### Release of PHL *in vitro*

200 μL of CT_0.3_P hydrogel was submerged in 2 mL of PBS and the release of PHL at 284 nm from external PBS (2 mL) was detected by an Ultraviolet-visible Spectrophotometer at specific time intervals.

### Shape adaptability and adhesion property of CTP hydrogel

The shape adaptability of the hydrogel was evaluated by dynamic porcine skin and static mold. Firstly, CT_0.3_P hydrogel was applied to the surface of the porcine skin and the skin was shaped by bending and twisting. Subsequently, the hydrogel was placed in molds of different shapes and the changes were recorded. Meanwhile, the hydrogel was adhered to the finger, and the residue was recorded after peeling. The adhesion property of the hydrogel was qualitatively assessed by visual adhesion experiments. And its adhesive strength was quantitatively analyzed by porcine skin shear lap test, in which commercial hydrogel (Hydrosorb Gel) was used as a control group. Refer to our previous article for specific experimental methods 28.

### Mechanical properties of CTP hydrogel

The rheological properties of the hydrogels, including storage modulus (G') and loss modulus (G''), were recorded using a rheometer (TA DHR2, USA). The hydrogel samples were made into cylinders with a diameter of 20 mm. The dynamic oscillation scanning frequency was 1-100 Hz, and the strain and temperature were set to 1% and 37°C, respectively. The tensile properties of hydrogels were performed using a universal tensile tester. The hydrogel was prepared into a rectangular shape with dimensions of 20 mm (length) × 10 mm (width) × 5 mm (thickness) and then subjected to tensile testing at a loading rate of 50 mm/min. The stress-strain curves of the samples were recorded.

### Antioxidant property *in vitro* of CTP hydrogel

The ability of hydrogel to scavenge H_2_O_2_ was assessed by H_2_O_2_ kit. Firstly, 200 μL hydrogel was added to 500 μL H_2_O_2_ (20 mM) solution and incubated at 37 °C for 1 h. The control group was replaced with DI for the hydrogel. The reaction solution was used to determine the H_2_O_2_ content according to the instructions of the H_2_O_2_ kit. The H_2_O_2_ scavenging rate of the hydrogel was calculated as follows: H_2_O_2_ scavenging rate (%) = (A_c_ - A_h_)/A_c_ × 100%, where A_h_ and A_c_ represent the absorbance values of the hydrogel and control groups, respectively. The hydrogel's ability to scavenge hydroxyl radicals was evaluated by the Safranin O assay 29. The hydrogel's ability to scavenge ABTS free radicals was assessed by the ABTS assay 30.

### Cellular level antioxidant property of CTP hydrogel

The protective effect of CTP hydrogel on cells under oxidative damage condition was evaluated by 2', 7'-dimethylfluorescein diacetate (DCFH-DA) fluorescent labeling technique. Firstly, culture medium containing 500 μM H_2_O_2_ was prepared. Subsequently, L929 was plated. When the cell fusion density reached 80%, the medium was replaced with medium containing H_2_O_2_ (500 μM). The experimental group added 20 µL hydrogel immediately after H_2_O_2_ treatment, while the positive group did not add hydrogel. After 1.5 h of incubation, the fluorescent probe DCFH-DA (10 μM) was added. After incubation for 25 min, the fluorescence images were captured by fluorescence microscope (Leica DFC7000T). Then the protective effect of CTP hydrogel on L929 under high glucose oxidative damage condition was further evaluated. This experiment was prepared instead with medium containing 500 μM H_2_O_2_ and 200 μM glucose. The rest of the steps were the same as the above experiment.

### Biocompatibility *in vitro* of CTP hydrogel

The blood compatibility of the hydrogel was evaluated by hemolysis assay, which was performed according to our previous report 31. The cytocompatibility of hydrogel on L929 cells was assessed by CCK-8 assay and live/dead cell staining. Sterile 100 μL hydrogel was immersed in 2 mL culture medium for 24 h to obtain the hydrogel extract. Then, L929 cells were cultured for 24 h after plating, and the medium was replaced with hydrogel extract solution. For the control group, fresh medium was continued to be used. The cells were then incubated for 24 h and 48 h. Cell viability and morphology at each time point were examined using CCK-8 assay and Calcein-AM/PI staining 32. Finally, cell survival and death images were taken using a fluorescence microscope.

### Cell migration and angiogenesis

The promotion effect of hydrogel on cell migration *in vitro* was evaluated by cell scratch assay. Firstly, 100 μL of sterile hydrogel was immersed in 2 mL of serum-free medium containing 500 μM H_2_O_2_, and the hydrogel extract solution was obtained 24 h later. Subsequently, human umbilical vein endothelial cells (HUVEC) were plated. When the fusion density reached 90%, uniform and consistent scratches were made. Then hydrogel extract solution was added, while serum-free medium containing 500 μM H_2_O_2_ was used as a control group. Then the scratched area was photographed at 0 h and 24 h. Finally, the changes in the width of the scratched area were analyzed by ImageJ software, and the cell migration rate was calculated by the formula: cell migration rate (%) = (W_a_-W_b_)/W_a_ × 100%, where W_a_ and W_b_ are the width of the 0 h and the 24 h cell scratches, respectively.

The ability of hydrogel to promote angiogenesis* in vitro* was evaluated by angiogenesis assay. Firstly, the hydrogel extract solution was prepared as described above. Then, Swe matrix gel was laid on the bottom of the well plate and waited for it to be solidified for cell plating, while medium containing 500 mΜ H_2_O_2_ was used as control group. After 4 h of incubation, 0.1% Calcein-AM was added. Finally, images were acquired using a fluorescence microscope, and the number of junctions, the area of blood vessels, the number of meshes and the total length of blood vessels were counted by ImageJ software.

### Photothermal and antibacterial properties of CTP hydrogel

The photothermal property of CTP hydrogel was evaluated by NIR laser (MDLIII-808 nm-2.5 W). Firstly, the hydrogel samples were irradiated under NIR (808 nm, 0.8 W/cm^2^), and the temperature changes were recorded and real-time thermal images were taken using a thermal imaging camera (T120, Guide). Then, the CT_0.3_P hydrogel was irradiated under NIR with different powers (0.6 W/cm^2^, 0.8 W/cm^2^, and 1.0 W/cm^2^) and the temperature changes were recorded.

The bacteriostatic activity *in vitro* of CT_0.3_P hydrogel was evaluated by *Escherichia coli* (*E. coli*, Gram-negative bacteria) and *Staphylococcus aureus* (*S. aureus*, Gram-positive bacteria). Firstly, 10 μL of *E. coli* or *S. aureus* solution (1 × 10^7^ CFU/mL) was added to the surface of the sterile hydrogel. Then, the hydrogel was exposed to NIR (808 nm, 0.8 W/cm^2^) for 0, 1, 5, 10, and 15 min, respectively. Meanwhile, the hydrogel was contacted with the bacterial suspension for 0, 1, 5, 10 and 15 min, respectively. The control group was untreated bacterial suspension. The bacteria were then resuspended in the medium for 24 h, and their absorbance values at 600 nm were determined after incubation. Subsequently, the bacterial suspension was diluted and plated on agar medium for further incubation. Finally, the colonization on the agar plates was photographed. The formula for calculating the antibacterial rate was: antibacterial rate (%) = (A_c_-A_h_)/(A_c_-A_b_) × 100%, where A_c_, A_h_ and A_b_ are the absorbance values of control, hydrogel and medium, respectively.

### Histocompatibility of CTP hydrogel

This study was approved by the Henan Academy of Pharmaceutical Sciences of Zhengzhou University (2024-YYY-049). KM male rats (SPF level) were provided by the Animal Experiment Center of Zhengzhou University and fed in the Animal Experiment Center. All experimental animals were handled in strict accordance with the relevant regulations of Zhengzhou University.

Biocompatibility *in vivo* was assessed by establishing a CT_0.3_P hydrogel subcutaneous embedding model. After shaving the dorsal hairs of the mice, 100 μL hydrogel was implanted under the skin. The control group was normal mice. On day 7 and day 14 after surgery, mice were euthanized and heart blood was taken for blood biochemical analysis. Subsequently, major organs of mice were taken after perfusion and stained for Hematoxylin and eosin (H&E).

### Blood coagulation index and hemostatic property of CTP hydrogel

The coagulation ability of hydrogel *in vitro* was evaluated by blood coagulation index (BCI). Firstly, 200 μL of hydrogel and an equal volume of gauze were placed in a well plate. Then, 10 μL of mouse blood was dropped on the surface of the hydrogel while 1 μL CaCl_2_ (0.2 M) solution was added and mixed. The control group was not treated with hydrogel. Then, the plate was incubated at 37 °C for 150 s and 2 mL of DI was added. Finally, the absorbance value of the solution at 540 nm was determined. The BCI was calculated by the formula: BCI (%) = (A_e_ - A_c_)/(A_c_ - A_d_) × 100%, where A_e_, A_c_, and A_d_ represent the absorbance values of the experimental group, the control group, and the DI, respectively.

The hemostatic effect *in vivo* of CT_0.3_P hydrogel was evaluated by liver bleeding model and tail bleeding model 33, 34. KM mice were randomly divided into three groups: control group, gauze group and CT_0.3_P group. Among them, the control group was not treated after surgery, while the gauze group was treated by applying gauze after surgery.

### Establishment and treatment of the diabetic mouse model of *S. aureus* infected wound

Diabetic model was established by streptozotocin (STZ). Firstly, KM mice were fasted without water restriction for 18 h before the experiment. Then, STZ was dissolved in pre-cooled sodium citrate buffer at a concentration of 1%. STZ was injected into the mice via intraperitoneal injection, and the mice were fasted without water restriction for 4 h. The non-fasting blood glucose value of more than 16.7 mmol/L after 14 days, accompanied by weight loss, excessive drinking, excessive urination, and excessive eating, was recognized as a successful diabetes model construction 35.

Next, a mouse model of full-thickness skin injury infection was constructed. Firstly, the anesthetized diabetic mice were dorsally depilated, and a full-thickness skin wound with a diameter of 9 mm was established. Subsequently, 50 μL *S. aureus* suspension (1×10^7^ CFU/mL) was added dropwise to the wound. After 5 h, the infected wound model was established. Later, the mice were randomly divided into four groups: control group (Hydrosorb Gel treatment), CT group (CT_0.3_ hydrogel treatment), CTP group (CT_0.3_P hydrogel treatment) and CTP + NIR group (CTP_0.3_P hydrogel combined with NIR treatment). Photographs were taken to record wound healing on days 0, 2, 4, 7, 10 and 14, and the wound area was calculated by ImageJ software. The wound healing rate was calculated as: wound healing rate (%) = (A_day0_-A_dayN_)/A_day0_ × 100%, where A_day0_ and A_dayN_ are the wound area on day 0 and day N, respectively.

### Antimicrobial property *in vivo* of CTP hydrogel

The bacteriostatic effect of each treatment group in infected skin wounds was assessed by co-culture method. On day 4, bacteria were collected from the wound area using a sterile cotton swab and then placed in liquid medium for 24 h. Then, the absorbance value of the bacterial suspension at 600 nm was determined. The formula for calculating the bacterial survival rate was: bacterial survival rate (%) = (A_e_-A_b_)/(A_c_-A_b_) × 100%, where A_e_, A_b_ and A_c_ are the absorbance values of the experimental group, liquid medium and control group, respectively. Meanwhile, the above bacterial solution was diluted and inoculated into agar plates. After 24 h, the growth of the colonies was photographed.

### H&E staining and Masson staining of skin wound area

Wound healing was assessed by histologic staining 36. On day 7 and day 14 after treatment, anesthetized mice were perfused. Subsequently, skin tissue from the wound area was collected and placed in paraformaldehyde. After fixation, the tissues were made into paraffin sections for H&E staining and Masson staining.

### Sirius red staining of skin wound area

The deposition and alignment of collagen fibers during wound healing were assessed by Sirius red staining. Firstly, skin tissue from the wound area was collected on day 7 after treatment, and paraffin sections were prepared and then dewaxed and dehydrated. Subsequently, they were stained dropwise with Sirius red staining solution. Next, the sections were sequentially dehydrated with gradient ethanol and transparent with xylene. Finally, the sections were sealed and the type and distribution of collagen fibers were observed under polarized light.

### Immunofluorescence

On day 7 and day 14 after treatment, skin tissues from the wound area were collected and fixed for 48 h, and then frozen sections and immunofluorescence were performed. The expression of scar-associated proteins (collagen type I, collagen type III, and YAP), inflammatory factors (cluster of differentiation 86, CD86; cluster of differentiation 206, CD206), and vascular markers (platelet endothelial cell adhesion molecule, CD31; alpha-smooth muscle actin, α-SMA) were detected, and images were taken with a fluorescence microscope.

### Transcriptome sequencing

On day 7 after treatment, wound tissues from diabetic mice were collected and washed with pre-cooled saline. Subsequently, the tissues were frozen and stored at -80 °C. RNA extraction, quality control, library construction and sequencing were performed by NovelBio Bio-Pharm Technology Co., Ltd. Differential gene screening was performed using the DESeq2 algorithm (threshold: Fold Change > 1.5 and FDR < 0.05).

### Statistical analysis

Differences between two groups were analyzed by t-test, differences between multiple groups were analyzed by One-way ANOVA, data were expressed as mean ± standard deviation (Mean ± SD), and the experiment was repeated at least three times. *P < 0.05, **P < 0.01, ***P < 0.001, ****P < 0.0001, indicate the level of statistical significance.

## Results and Discussion

### Preparation, swelling property, FTIR analysis and internal structure characterization of CTP hydrogel

**Figure 2:**
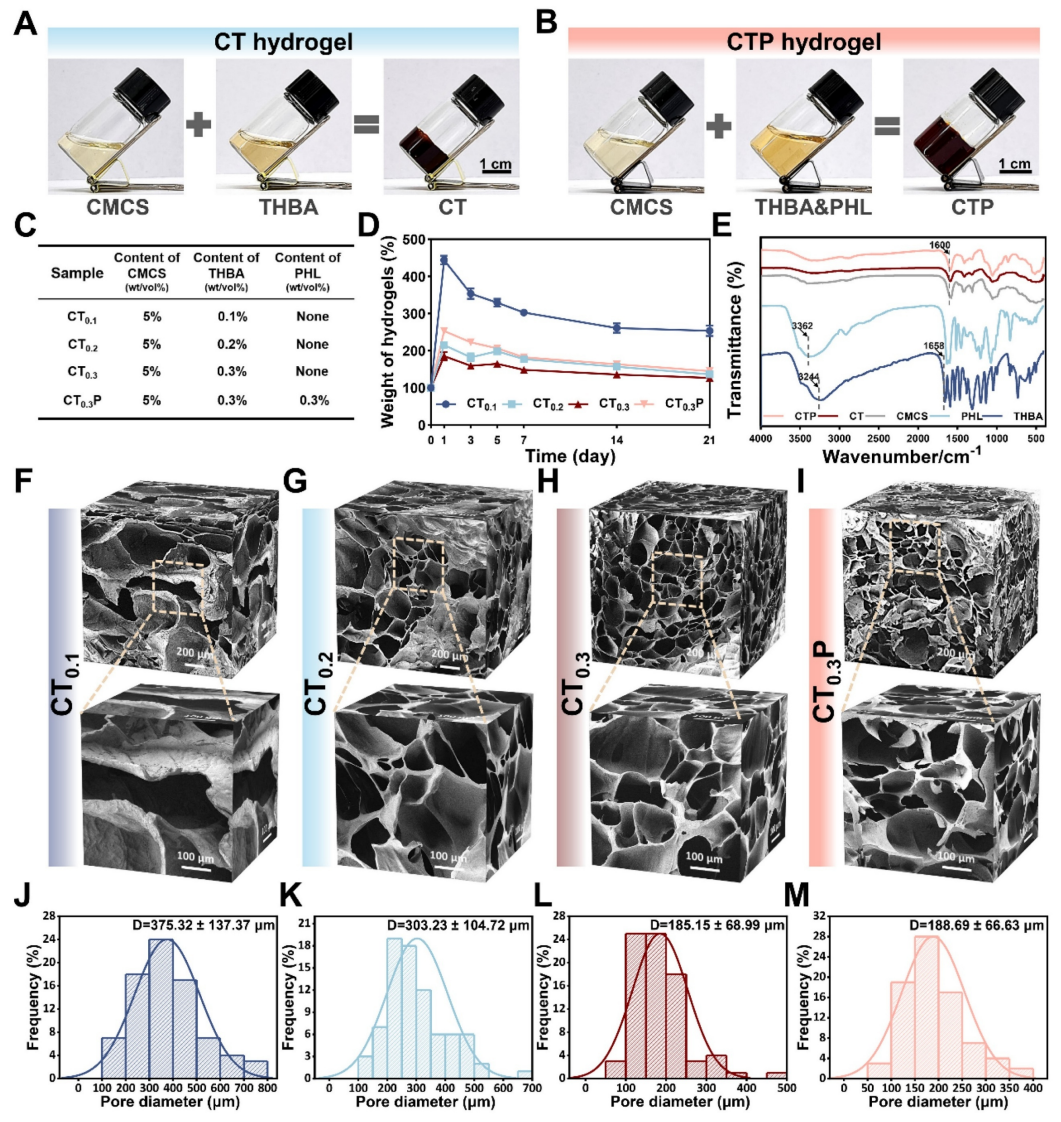
Preparation and characterization of CTP hydrogels. (A) Graphs of the gelation process of CT hydrogel. (B) Graphs of the gelation process of CTP hydrogel. (C) Composition and content of CT_0.1_, CT_0.2_, CT_0.3_, and CT_0.3_P hydrogels. (D): Swelling curves. (E) FTIR of CMCS, THBA, PHL, CT and CTP hydrogels. (F-M) SEM images of the hydrogels and corresponding pore size distributions (mean ± SD, n = 3).

The one-pot method was used to synthesize CTP hydrogel, which is an efficient and easy gelation strategy. In the preparation of CT hydrogel, equal volumes of CMCS and THBA solutions were mixed thoroughly, and the formed gel was attached to the bottom of the bottle with no flowability (Figure 2A). CTP hydrogel, on the other hand, was formed by the reaction of CMCS with a mixed solution of THBA&PHL (Figure 2B). Among them, the low dissolution of PHL caused it to exhibit low bioavailability under physiological conditions. In our study, we found that it could be completely dissolved by the ultrasound-heating cycle method, thus laying a certain foundation for improving the bioavailability of PHL (Figure S1). Through the preliminary exploration, we determined 3 mg/mL as the optimal concentration of PHL to promote cell migration (Figure S2). Meanwhile, four formulations, CT_0.1_, CT_0.2_, CT_0.3_, and CT_0.3_P, were set up in order to investigate the effects of THBA content and the introduction of PHL on the properties of hydrogel (Figure 2C). Of these, CT_0.1_, CT_0.2_, and CT_0.3_ differed in THBA content, while CT_0.3_P added PHL to the CT_0.3_ formulation. Ideal wound dressings should have the ability to absorb wound exudate efficiently to accelerate the wound healing process 37. The results of the swelling curves showed that the swelling rates of all hydrogels peaked on day 1, reaching 443.95%, 215.02%, 185.74% and 253.25% for CT_0.1_, CT_0.2_, CT_0.3_ and CT_0.3_P, respectively. It showed that the hydrogel was able to rapidly absorb tissue fluid from the wound site (Figure 2D).

Furthermore, the variation of functional groups in CMCS, THBA, PHL, CT and CTP hydrogels was analyzed by FTIR (Figure 2E). In THBA, the broad absorption peak at 3244 cm^-1^ and the weak absorption peak at 1658 cm^-1^ were attributed to the stretching vibrations of -OH and -C=O, respectively 38. In the FTIR spectrum of PHL, the absorption peak at 3362 cm^-1^ was attributed to -OH 39. In addition, CT and CTP hydrogels showed broadened absorption peaks at 1600 cm^-1^ compared to CMCS due to the overlap of -C=N formed by -NH_2_ in CMCS and -C=O in THBA with the -C=O stretching vibration peak of the amide group on CMCS 40. The loose porous structure endowed the hydrogel with excellent water absorption and air permeability, and also improved the drug loading capacity of the hydrogel 27. Results of SEM showed that all hydrogels exhibited a loose porous structure. With the increase of THBA content, the cross-linking of the hydrogels became denser and the pore size gradually became smaller (Figure 2F-M). This porous structure is similar to the natural extracellular matrix, which facilitates cell adhesion and proliferation, further promoting tissue repair 41. Specifically, the average pore sizes of CT_0.1_, CT_0.2_, CT_0.3_, and CT_0.3_P hydrogels were 375.32 ± 137.37 μm, 303.23 ± 104.72 μm, 185.15 ± 68.99 μm, and 188.69 ± 66.63 μm, respectively (Figure 2K-N).

### Adhesion property and shape adaptability of CTP hydrogel

**Figure 3:**
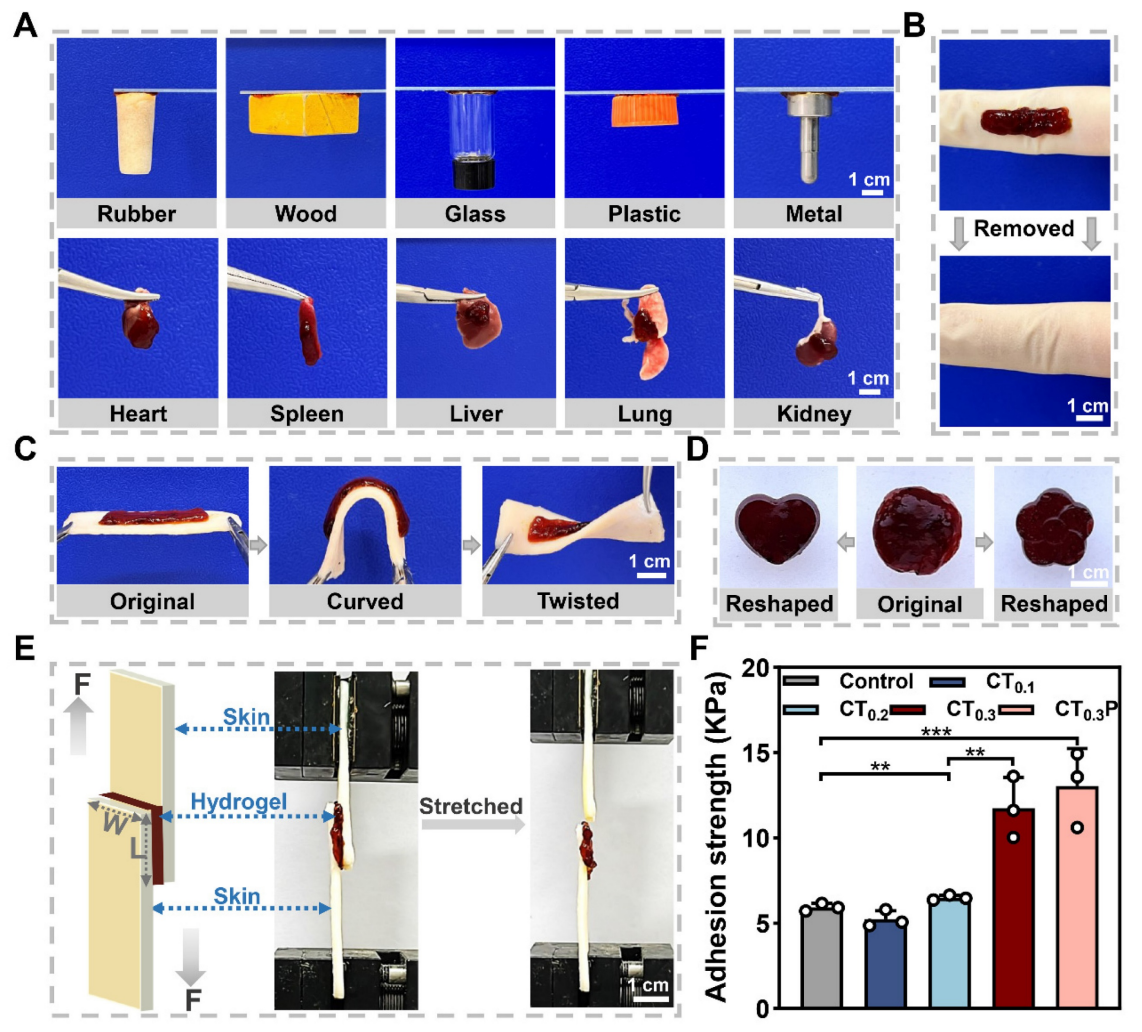
Adhesion property and shape adaptability of CTP hydrogels. (A) Photographs of CT_0.3_P hydrogel adhered to different materials. (B) Photographs before and after the removal of CT_0.3_P hydrogel. (C) Adhesion property of CT_0.3_P hydrogel to porcine skin. (D) Initial image of the hydrogel's shape and the images of the shape after mold processing. (E) Schematic diagrams and photographs of the lap shear experiments, F is the force, and W is width, L is length. (F) Maximum adhesion strength of hydrogel to porcine skin (**P < 0.01, ***P < 0.001, mean ± SD, n = 3).

Good adhesion property allows hydrogel to integrate tightly with tissue and continuously provide a beneficial microenvironment similar to extracellular matrix for wound 42, 43. The adhesion property of CT_0.3_P hydrogel was first qualitatively tested. The results showed that the CT_0.3_P hydrogel was able to firmly adhere to a wide range of non-biological and biological tissues (Figure 3A). As a skin wound dressing, hydrogel not only need to have sufficient adhesion, but also should be easy to remove from the wound in order to avoid secondary damage 44. Therefore, the removal of hydrogel from the skin was further investigated. The results showed that the CT_0.3_P hydrogel was able to be removed from the skin without leaving a stain (Figure 3B). Meanwhile, the hydrogel remained adherent even after bending and twisting during the dynamic adaptation with the porcine skin (Figure 3C). In addition, the original hydrogel could be fully adapted to flower-shaped and heart-shaped molds, indicating that the CT_0.3_P hydrogel had the ability to adaptively adjust to the shape and size of the wound (Figure 3D). Moreover, the storage modulus of each hydrogel was greater than the loss modulus. The storage modulus is a key index of hydrogel elasticity, indicating that all groups of hydrogels have a certain degree of elasticity. Meanwhile, the addition of PHL significantly increased the elongation at break of the hydrogel, thus improving the ductility of the hydrogel (Figure S3).

To quantitatively assess the adhesion property of the hydrogels, a shear lap test was performed using porcine skin as the experimental tissue (Figure 3E). The results showed that the adhesion strengths in the control (Hydrosorb Gel), CT_0.1_, CT_0.2_, CT_0.3_ and CT_0.3_P hydrogel groups were 5.94 ± 0.23, 5.25 ± 0.49, 6.50 ± 0.14, 11.74 ± 1.79, and 13.03 ± 2.21 kPa, respectively (Figure 3F). The good tissue adhesion of the hydrogels was attributed to the presence of polyphenolic structures in THBA, where polyphenolic groups bind to biological tissues through a variety of interactions that exhibit high tissue affinity, including hydrogen bonding, electrostatic interaction, metal-ligand interaction, π-π stacking interaction, and covalent cross-linking formed after oxidation to quinone 45, 46. Meanwhile, the adhesion of the hydrogels was gradually enhanced with increasing THBA concentration, and the adhesion strength of both CT_0.3_ and CT_0.3_P hydrogels was significantly higher than that of the control. These experimental results suggest that CT_0.3_P hydrogel has good tissue adhesion and can flexibly adapt to the wound shape and avoid wound infection.

### Antioxidant property of CTP hydrogel *in vitro*

Diabetic wounds are mainly characterized by increased levels of reactive oxygen species and the persistence of oxidative stress 47. Oxidative stress is usually accompanied by the overproduction of free radicals, including hydroxyl radical, ABTS radical, and H_2_O_2_, which are susceptible to causing damage to organisms and triggering apoptosis 48. Antioxidant hydrogel can balance the oxidative stress at the wound site by scavenging excess ROS, thus promoting the wound healing process 49.

**Figure 4:**
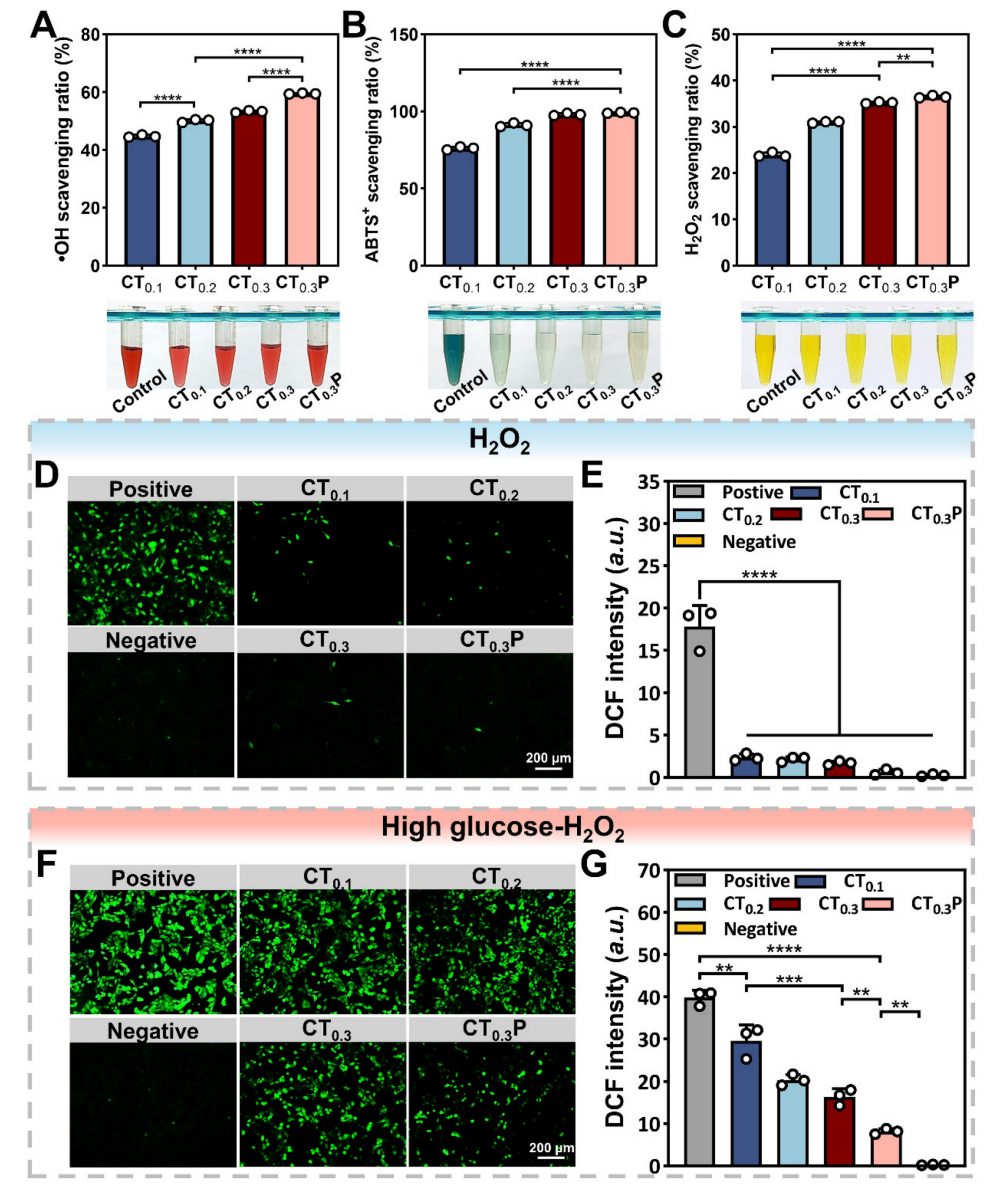
Antioxidant property of CTP hydrogel *in vitro*. Statistical graphs and photographs of the scavenging rate of CT_0.1_, CT_0.2_, CT_0.3_ and CT_0.3_P hydrogels against A) hydroxyl radicals, B) ABTS radicals and C) H_2_O_2_. (D) DCF fluorescence staining images of L929 cells treated with 500 µM H_2_O_2_ (Positive group), 500 µM H_2_O_2_ + 20 µL CT_0.1_ hydrogel (CT_0.1_ group), 500 µM H_2_O_2_ + 20 µL CT_0.2_ hydrogel (CT_0.2_ group), 500 µM H_2_O_2_ + 20 µL CT_0.3_ hydrogel (CT_0.3_ group) and 500 µM H_2_O_2_ + 20 µL CT_0.3_P hydrogel (CT_0.3_P group), without H_2_O_2_ (Negative group). (E) DCF fluorescence statistics of different groups under oxidative damage condition. (F) DCF fluorescence staining images after treatment of L929 cells with 500 µM H_2_O_2_ + 200 µM glucose (Positive group), 500 µM H_2_O_2_ + 200 µM glucose + 20 µL CT_0.1_ hydrogel (CT_0.1_ group), 500 µM H_2_O_2_ + 200 µM glucose + 20 µL CT_0.2_ hydrogel (CT_0.2_ group), 500 µM H_2_O_2_ + 200 µM glucose + 20 µL CT_0.3_ hydrogel (CT_0.3_ group) and 500 µM H_2_O_2_ + 200 µM glucose + 20 µL CT_0.3_P hydrogel (CT_0.3_P group), without H_2_O_2_ (Negative group). (G) DCF fluorescence statistics of different groups under high glucose oxidative damage condition (**P < 0.01, ***P < 0.001, ****P < 0.0001, mean ± SD, n = 3).

The scavenging ability of CTP hydrogel on hydroxyl radical was determined by using the Safranin O assay. The results showed that the scavenging ratios of hydroxyl radical by CT_0.1_, CT_0.2_, CT_0.3_ and CT_0.3_P hydrogels were 44.79%, 50.09%, 53.27% and 59.49%, respectively. (Figure 4A). The scavenging ability of hydrogel on ABTS radical was determined by ABTS method. The results showed that the scavenging ratios of CT_0.1_, CT_0.2_ and CT_0.3_ hydrogels against ABTS radical were 80.29%, 92.79% and 97.98%, respectively, while the scavenging ratio of CT_0.3_P hydrogel was as high as 99.10% (Figure 4B). Notably, all reactions were completed within 5 min, indicating that CTP hydrogel has the ability to scavenge ABTS radicals quickly and efficiently. Excess H_2_O_2_ triggers oxidative stress, which in turn inhibits cell proliferation and tissue repair 50. The results showed that the scavenging activity of CT_0.3_ hydrogel on H_2_O_2_ was about 35.21%, which was about 1.5 times higher compared to CT_0.1_ hydrogel treatment (Figure 4C). Due to the unique chemical structure and multiple mechanisms of action that endowed PHL with excellent antioxidant capacity, the introduction of PHL enabled the CT_0.3_P hydrogel to exhibit the strongest scavenging effect on H_2_O_2_. The above results showed that the CTP hydrogel had excellent scavenging ability for hydroxyl radical, ABTS radical and H_2_O_2_, and the scavenging rate increased with the growing of THBA concentration and the introduction of PHL.

The ROS scavenging activity of CTP hydrogel was next analyzed at the cellular level. The results showed that cells in the positive group exhibited intense green fluorescence, indicating a significant increase in intracellular ROS levels. While in hydrogel groups, the intensity of intracellular fluorescence was significantly weakened (Figure 4D). The quantitative analysis results further confirmed this trend, and the CT_0.3_P hydrogel exhibited the best intracellular ROS scavenging activity (Figure 4E). In diabetic wounds, the high glucose environment aggravates ROS production, which leads to wound deterioration 51, 52. Under high glucose oxidative damage condition, CTP hydrogel still exhibited significant ROS scavenging activity (Figure 4F). In addition, the ROS scavenging ability was enhanced with the growing of THBA content and the introduction of PHL. In particular, CT_0.3_P hydrogel significantly inhibited intracellular ROS production, resulting in a 3.6 times reduction in intracellular ROS levels compared to CT_0.1_ hydrogel (Figure 4G). These results suggest that CTP hydrogel can effectively protect cells from oxidative stress under oxidative damage and high glucose oxidative damage conditions.

### Biocompatibility of CTP hydrogel

**Figure 5:**
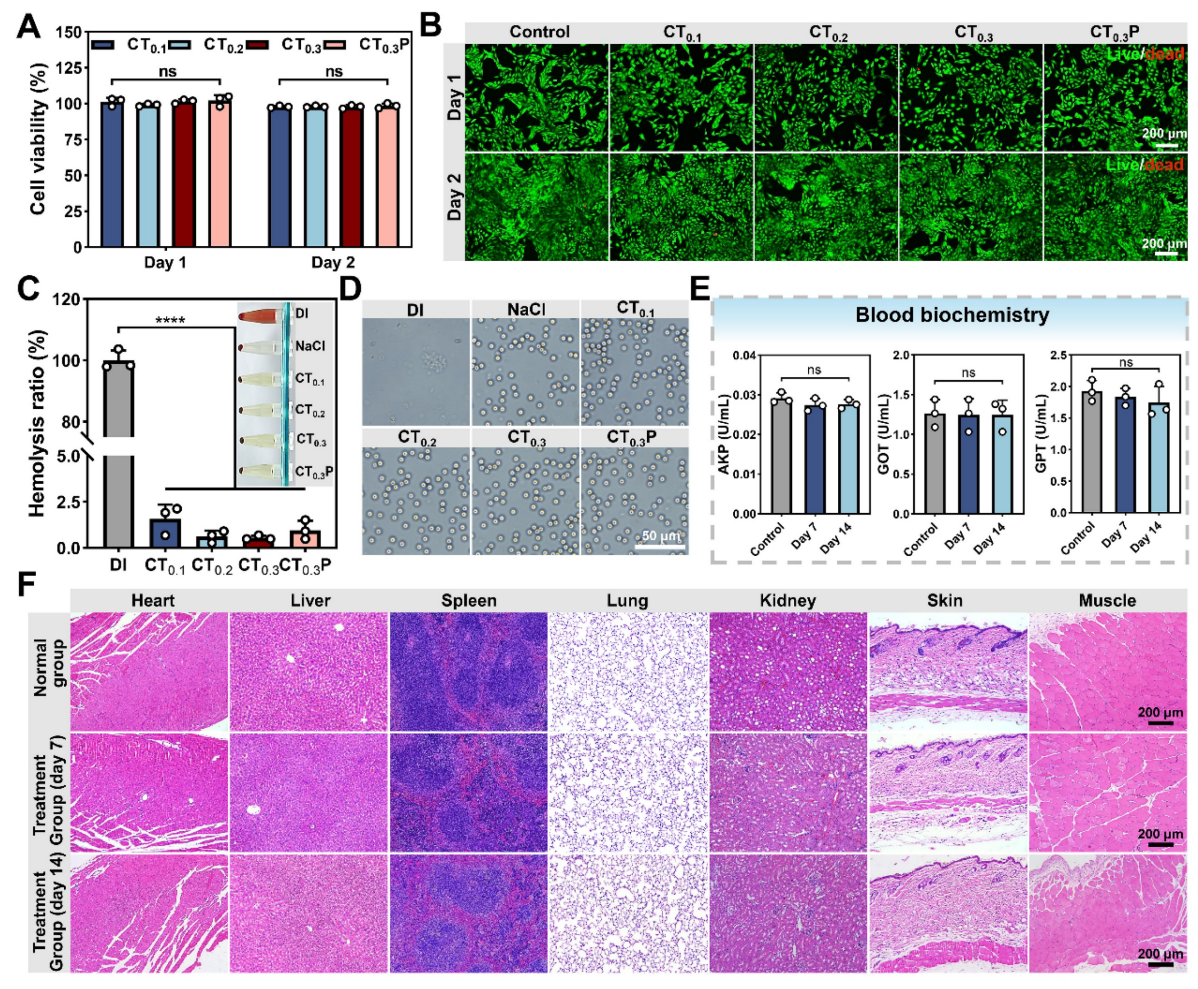
Biocompatibility of CTP hydrogel. (A) Statistical graphs of cell viability of L929 cells cultured in hydrogel extraction on day 1 and day 2. (B) Live/dead fluorescent double-stained images on day 1 and day 2. (C) Statistical graphs of hemolysis ratio and hemolysis photograph (inset). (D) Photographs of red blood cells morphology. (E) Activity statistic graphs of AKP, GOT, and GPT. (F) H&E staining images of major organs, muscles and skin around the wounds of CT_0.3_P hydrogel implanted mice and normal mice on day 7 and day 14 (ns: not statistically significant, ****P < 0.0001, mean ± SD, n = 3).

Good biocompatibility is the basis for the widespread use of hydrogel as wound dressing 53. After 1 and 2 days of co-culture with hydrogel extract, the cell viability in CT_0.1_, CT_0.2_, CT_0.3_, and CT_0.3_P hydrogel groups was 101.25% and 97.78%, 99.04% and 97.87%, 101.51% and 97.73%, 102.20% and 98.42%, respectively (Figure 5A), which satisfied 70% of the international biomaterial standard (ISO10993:2009) 54. The results in live/dead fluorescence double staining experiment were consistent with the data of cell viability test, and the majority of L929 cells were labeled with bright green fluorescence and presented the complete shuttle-like shape (Figure 5B). It indicated that the L929 cells treated with hydrogel extracts still maintained high cell viability. Therefore, CTP hydrogel has excellent good cytocompatibility.

The blood compatibility of CTP hydrogel was evaluated by hemolysis experiment. The results showed that after 1 h of co-incubation of hydrogels with blood, the hemolysis rates of all four hydrogel groups were less than 5% (Figure 5C), which were in accordance with international standard 55. Subsequently, the morphology of red blood cells (RBCs) in each group was further observed. The RBCs in all hydrogel groups showed no visible damage and the morphology was similar with that of the saline group (Figure 5D). These results indicated that CTP hydrogel has good blood compatibility, showing its great potential application as a wound dressing.

After implanting CT_0.3_P hydrogel into the dorsum of mouse, H&E staining results showed that as compared with the normal group, no obvious inflammatory reaction was found in the major organs in the CT_0.3_P hydrogel group both on day 7 and day 14. Meanwhile, the skin and muscle tissues around the wound did not show any pathological changes or systemic damage (Figure 5F). The activities of serum biomarkers (AKP, GOT, GPT) were also analyzed in the normal and hydrogel groups on day 7 and day 14. The results showed that the activities of each index in the hydrogel group were not significantly different from the normal group (Figure 5E). It indicated that CT_0.3_P hydrogel had no significant toxic effect on liver function 56. In summary, the experimental findings showed that the CT_0.3_P hydrogel possessed good biocompatibility, which provided strong support for subsequent animal experiments.

### Cell migration and angiogenesis

**Figure 6:**
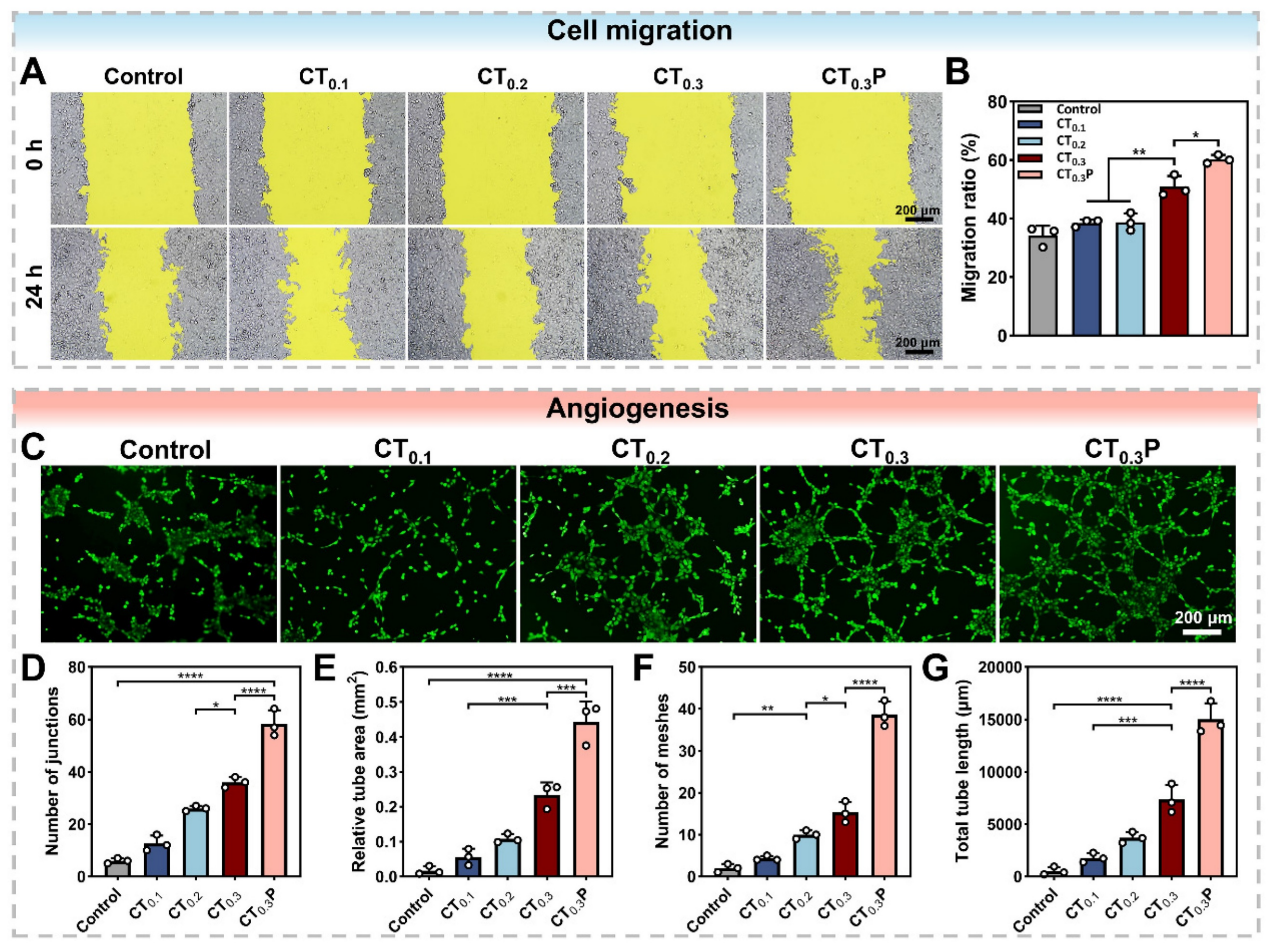
CTP hydrogel promotes cell migration and angiogenesis *in vitro*. (A) Cell migration images of HUVEC at 0 h and 24 h under different treatment conditions. (B) Statistical graph of cell migration rates. (C) Angiogenesis images of HUVEC after 4 h under different treatments; Statistical graphs of D) the number of junctions, E) the area of tubes, F) the number of meshes, and G) the total length of blood vessels in the control, CT_0.1_, CT_0.2_, CT_0.3_, and CT_0.3_P groups (*P < 0.05,**P < 0.01, ***P < 0.001, ****P < 0.0001, mean ± SD, n = 3).

Cell migration is the basis of angiogenesis and fiber proliferation 57. The promotion of HUVEC migration by CTP hydrogel under oxidative stress condition was evaluated by cell scratch assay. After 24 h of co-culture with the hydrogel extract, compared with the control group, the scratched areas were significantly reduced in the CT_0.3_ and CT_0.3_P groups, indicating that the CT_0.3_ and CT_0.3_P groups were still able to promote cell migration under oxidative stress condition (Figure 6A). In particular, the CT_0.3_P group significantly increased the cell migration rate from 50.92% to 60.26% compared with the CT_0.3_ group (Figure 6B). It demonstrated that the introduction of PHL could enhance the ability of hydrogel to promote cell migration under oxidative stress condition.

Impaired vascular function is a key obstacle in the diabetic wound healing process. Neovascularization accelerates the wound repair process through multiple mechanisms, such as providing nutrients and oxygen, modulating inflammatory response and supporting granulation tissue formation 58. Swe matrix gel was used to simulate the extracellular matrix to further investigate the ability of CTP hydrogel to promote angiogenesis under oxidative stress condition. Tubular network structures were clearly observed in hydrogel-treated groups compared to control group. It might be attributed to the strong antioxidant ability of the hydrogel, which provided a favorable microenvironment for angiogenesis. In particular, the CT_0.3_P hydrogel group exhibited a more developed tubular network structure (Figure 6C). Quantitative analysis showed that the CT_0.3_P hydrogel group had the highest number of junctions, vessel area, and number of meshes, and the longest total vessel length compared with control, CT_0.1_, CT_0.2_, and CT_0.3_ groups (Figure 6D-G). Therefore, CT_0.3_P hydrogel possesses the ability to promote angiogenesis under oxidative stress condition and has potential application in diabetic wound therapy.

### Photothermal property and antimicrobial property

**Figure 7:**
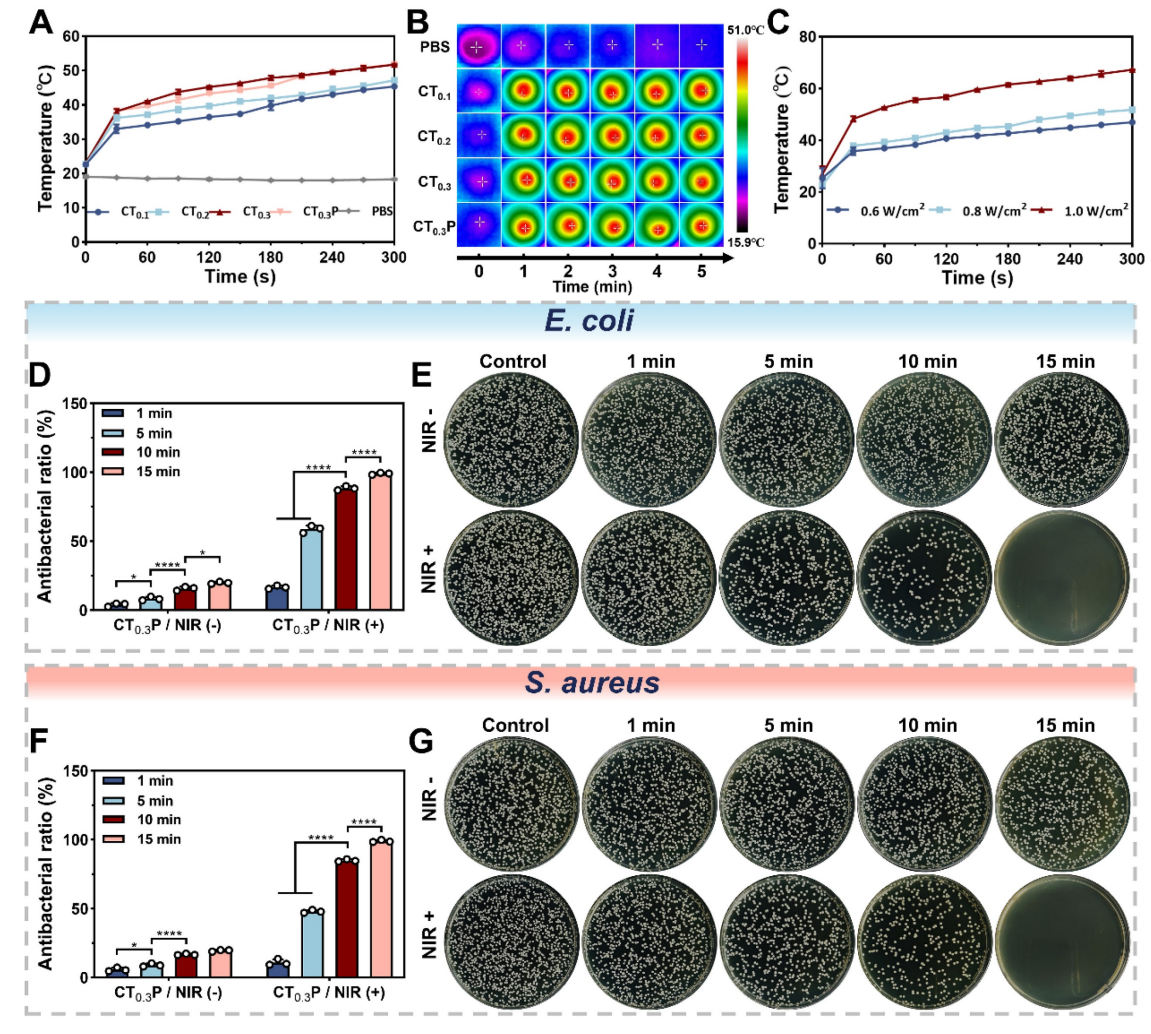
Photothermal and antimicrobial properties of CTP hydrogel. (A) Temperature change curves of NIR (808 nm, 0.8 W/cm^2^) irradiated PBS, CT_0.1_, CT_0.2_, CT_0.3_, and CT_0.3_P hydrogels during 300 s.(B) Real-time thermal images of PBS, CT_0.1_, CT_0.2_, CT_0.3_, and CT_0.3_P hydrogels during NIR irradiation for 300 s. (C) Temperature curves of CT_0.3_P hydrogel irradiated by NIR for 300 s at different powers (0.6 W/cm^2^, 0.8 W/cm^2^, 1.0 W/cm^2^). (D) Statistical graphs of the antibacterial rates against *E. coli* after 0, 1, 5, 10 and 15 min of CT_0.3_P hydrogel without and with NIR irradiation. (E) Photographs of the corresponding colonies of the antimicrobial effect of CT_0.3_P hydrogel on *E. coli*. (F) Statistical graphs of the antimicrobial rates against *S. aureus* after 0, 1, 5, 10 and 15 min of CT_0.3_P hydrogel without and with NIR irradiation. (G) Photographs of the corresponding colonies of CT_0.3_P hydrogel with antimicrobial effect against* S. aureus* (*P < 0.05, ****P < 0.0001, mean ± SD, n = 3).

Photothermal hydrogel is able to efficiently convert light energy into thermal energy, thus achieving localized warming by NIR irradiation 59. To evaluate the photothermal property, CTP hydrogel was irradiated with NIR (0.8 W/cm^2^, 808 nm). The results showed that after exposure for 5 min, the CT_0.1_, CT_0.2_, CT_0.3_, and CT_0.3_P hydrogels reached temperatures of 45.37 °C, 47.2 °C, 51.8 °C, and 51.8 °C, respectively (Figure 7A). The temperature of the hydrogels gradually increased with the increase of THBA content, which indicated that the photothermal effect of CTP hydrogels was closely related to the content of THBA. We deduced that this could be attributed to the polyphenol oxidation in THBA which in turn conferred the hydrogel with NIR light absorption and photothermal ability. Real-time thermal images showed that under NIR laser exposure, the temperature change of PBS was almost negligible, while the hydrogel groups exhibited significant photothermal response (Figure 7B). In addition, the maximum temperature of the hydrogels increased from 46.93 °C to 67.27 °C when the laser power density was increased (Figure 7C), indicating that the CT_0.3_P hydrogel has strong photothermal conversion ability. Therefore, the laser power density of 0.8 W/cm^2^ was set for subsequent experiments.

The persistent high glucose environment of diabetic wound is susceptible to bacterial infection, which can trigger a sustained local inflammatory response and destroy new tissues 60. The results showed that the bacterial inhibition rates of the CTP hydrogel without photothermal treatment were consistently lower than 20% against *E. coli* and *S. aureus*, suggesting that the inherent antibacterial ability of the hydrogel for a short period of time is insufficient to completely eliminate bacteria. In contrast, for both *E. coli* and *S. aureus*, the inhibition rates of CT_0.3_P hydrogel gradually increased with the prolongation of NIR (0.8 W/cm^2^, 808 nm) exposure time. After 15 min, the inhibition rates of CT_0.3_P hydrogel against* E. coli* and *S. aureus* reached 98.95% and 98.86%, respectively, which almost completely killed the bacteria (Figure 7D and Figure 7F). When the temperature was increased to 45 °C it would trigger the denaturation of bacterial enzymes, which in turn would disrupt the cell membrane and ultimately lead to bacterial death 61. In addition, the growth of bacteria on the plate was consistent with the above results (Figure 7E and Figure 7G). These results suggest that the inherent bacterial inhibition of CT_0.3_P hydrogel synergistically with photothermal therapy demonstrated excellent antibacterial performance, which is expected to play an important role in the treatment of infected wounds.

### Hemostatic property of CTP hydrogel

**Figure 8:**
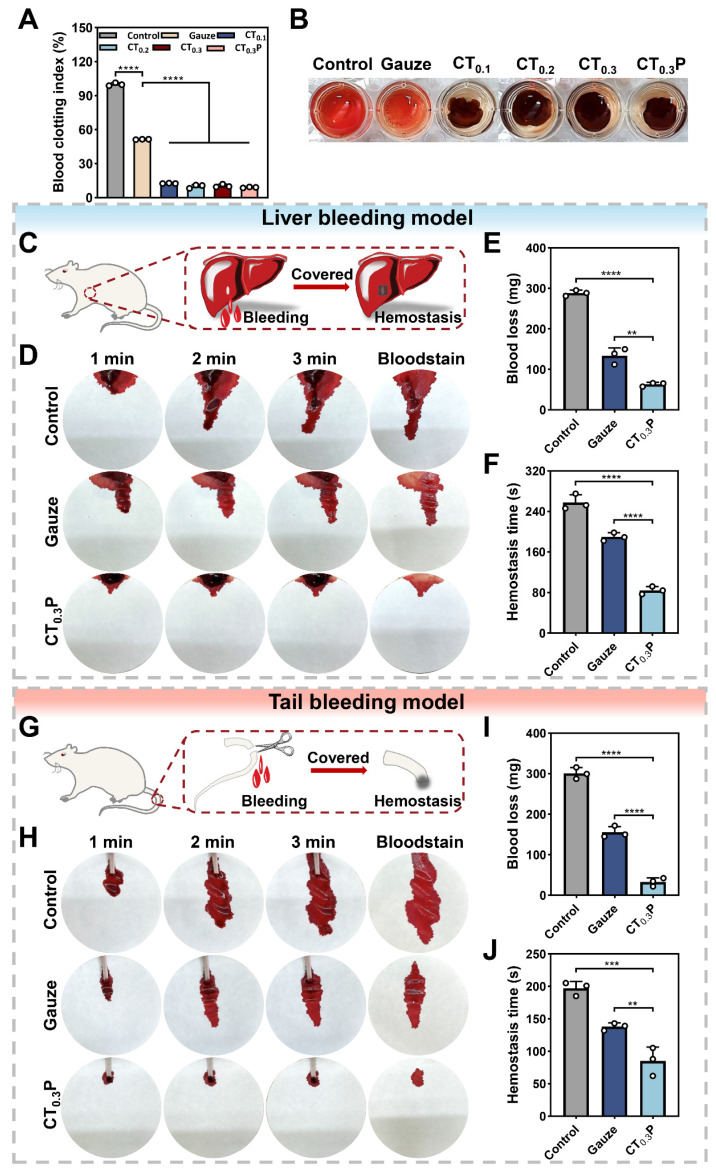
Hemostatic properties of CTP hydrogel. (A) Statistical graphs of BCI index in control, gauze, CT_0.1_, CT_0.2_, CT_0.3_, and CT_0.3_P hydrogel groups. (B) Photographs of coagulation assay. (C) Schematic diagram of mouse liver hemorrhage model. (D) Photographs of liver hemorrhage within 3 min under different treatments. (E) Statistical graphs of blood loss from liver. (F) Statistical graphs of hemostasis time of liver. (G) Schematic diagram of the mouse tail hemorrhage model. (H) Photographs of tail hemorrhage within 3 min under different treatments. (I) Statistical graph of tail blood loss. (J) Statistical graph of tail hemostasis time (**P < 0.01, ***P < 0.001, ****P < 0.0001, mean ± SD, n = 3).

Coagulation plays a key role in wound healing, in addition to rapidly stopping bleeding, it also effectively prevents infection, initiates inflammatory response, promotes tissue repair and regulates scar formation 62. The coagulation performance of CTP hydrogel *in vitro* was evaluated by blood coagulation index (BCI). The results showed that the BCI of the control and gauze groups were 100.00% and 51.48%, while the BCI of CT_0.1_, CT_0.2_, CT_0.3_, and CT_0.3_P hydrogels were 12.52%, 9.99%, 10.41%, and 9.00%, respectively (Figure 8A). In addition, it was observed that RBCs in the control and gauze groups fractured and the supernatants appeared red in color. In contrast, the supernatant in the hydrogel group was lighter in color, indicating that most of the blood had coagulated (Figure 8B). The above results indicate that CTP hydrogel has a good coagulation ability.

Hemostasis, as the initiation of wound healing, is critical to the overall wound repair process 63. The hemostatic ability of CT_0.3_P hydrogel was evaluated by liver bleeding model and tail bleeding model. In the liver bleeding model, CT_0.3_P hydrogel completely stopped bleeding after 84 s of treatment, whereas the other groups showed varying degrees of bleeding. Both the gauze group (133.57 mg) and the CT_0.3_P group (63.10 mg) exhibited less blood loss after 120 s compared to the control group (288.37 mg) (Figure 8C-F). In the tail bleeding model, the control group showed a large amount of blood on the filter paper within 3 min, while the CT_0.3_P group showed significantly less blood (Figure 8G-H). Further quantitative results showed that the amount of blood in the CT_0.3_P group (32.73 mg) was significantly lower than that in the gauze group (155.43 mg) and the control group (300.47 mg). The hemostasis time also showed a similar trend (Figure 8I-J). The above experimental data showed that CT_0.3_P hydrogel exhibited strong hemostatic effect in both models. The reasons for the excellent hemostatic performance of CT_0.3_P hydrogel might include: (1) the adhesive property of the hydrogel can form a physical barrier to cover the wound surface 64; (2) the porous structure and swelling property of the hydrogel can effectively absorb exudate at the wound site and promote clotting factor accumulation in the wound area 53; (3) The -NH_2_ group of CMCS can promote platelet attachment and aggregation through electrostatic interactions 65; (4) The phenolic groups in the hydrogel are also capable of strong interactions (hydrogen bonding and π-π interactions) with components of the blood, which in turn promote hemostasis 56. These results suggest that CT_0.3_P hydrogel has excellent *in vivo* hemostatic ability and has potential application in the treatment of diabetes-induced hemorrhagic wound repair.

### Establishment and treatment of *S. aureus* infected diabetic wound models

**Figure 9:**
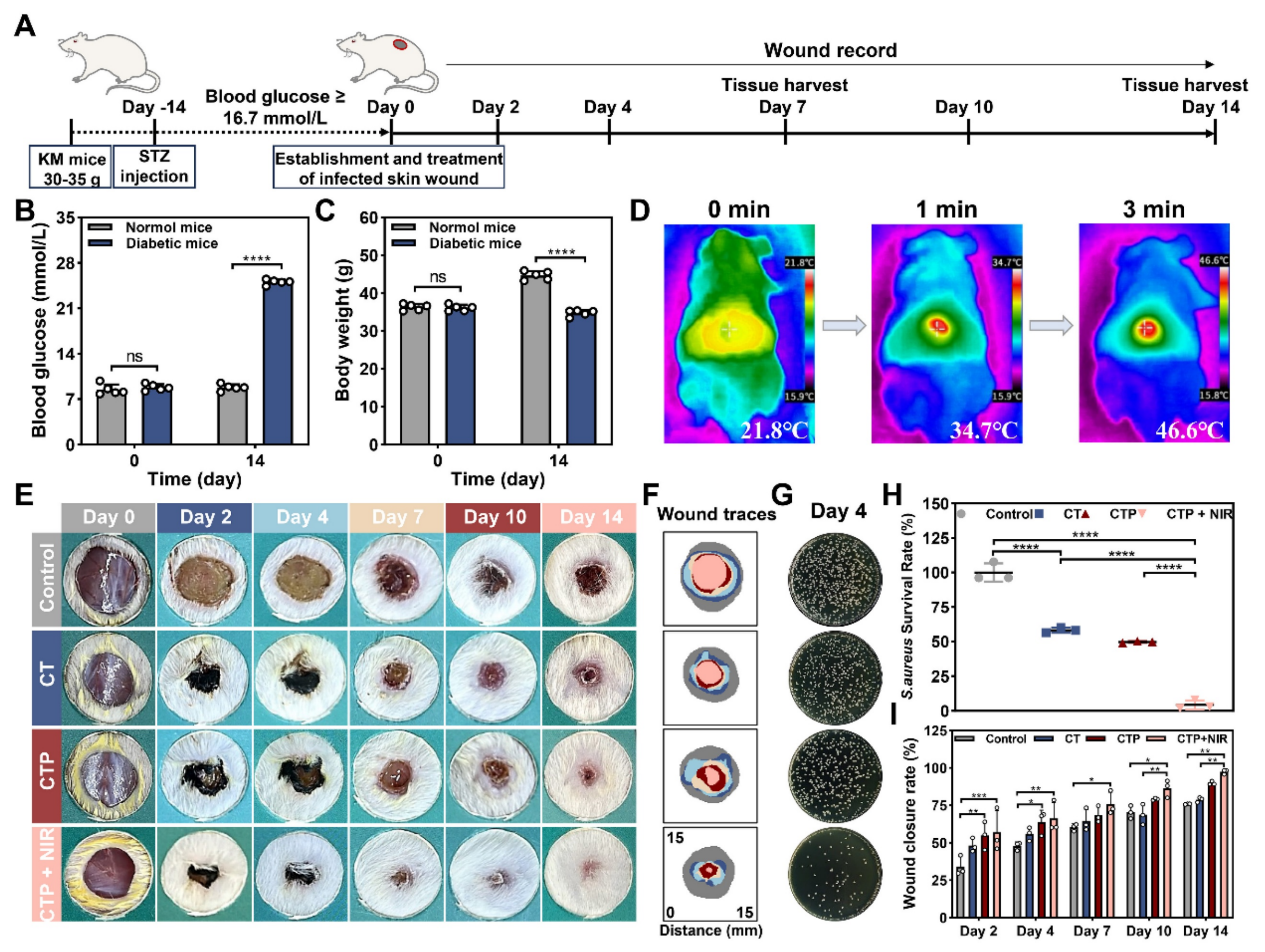
Establishment and treatment of diabetic wound model infected by *S. aureus*. (A) Schematic diagram of the establishment and treatment of infected diabetic wound model. (B) Statistical graph of blood glucose changes in mice before and after STZ injection. (C) Statistical graph of the changes in body weight of mice before and after STZ injection. (D) Thermal images of CT_0.3_P hydrogel *in vivo* under NIR exposure. (E) Photographs of wound healing in each group on days 0, 2, 4, 7, 10 and 14. (F) Tracer graphs of wound healing corresponding to each group. (G) Photographs of colonies of bacteria at the wound on day 4. (H) Statistical graph of bacterial survival rates in each group. (I) Statistical graph of wound healing rates (ns: not statistically significant, *P < 0.05, **P < 0.01, ***P < 0.001, ****P < 0.0001, mean ± SD, n ≥ 3).

After considering the properties of adhesion, antioxidant, biocompatibility, cell migration, angiogenesis, photothermal antibacterial, and hemostasis, CT_0.3_P hydrogel was selected to evaluate its effect in the infected diabetic mouse model of full-thickness skin injury. The animal experimental procedure was as follows: the diabetic model was constructed by streptozocin (STZ). Then a full-thickness skin injury model of *S. aureus* infection was established on the dorsum of diabetic mice, and the wounds were treated with commercial hydrogel Hydrosorb Gel (control group), CT_0.3_ hydrogel (CT group), CTP hydrogel (CTP group), and CT_0.3_P hydrogel combined with NIR (CTP + NIR group), respectively. Wound healing was recorded on days 0, 2, 4, 7, 10, and 14, and tissues from the wound areas were collected on days 7 and 14 (Figure 9A). The results showed that on day 14 after STZ injection, compared with normal mice, diabetic mice had a blood glucose level of more than 16.7 mmol/L and a significant decrease in body weight, indicating that the diabetic model was successfully constructed (Figure 9B-C). Meanwhile, we evaluated the photothermal property* in vivo* of CT_0.3_P hydrogel. The initial temperature of CT_0.3_P hydrogel irradiated by NIR was 21.8 °C, and the temperature increased to 46.6 °C after 3 min, which indicated that CT_0.3_P hydrogel also had efficient photothermal property *in vivo* (Figure 9D). From the wound healing photograph at each time point, all groups showed gradual decrease in wound area over time. However, on day 14, larger wound areas were still visible in the control and CT groups. Notably, on day 2, the wound area in the CTP + NIR group decreased rapidly as the hydrogel contracted under NIR exposure during treatment, significantly improving the wound healing rate at an early stage (Figure 9E-F).

Considering the strong antimicrobial property* in vitro* of CT_0.3_P hydrogel, we collected bacterial samples from the wound on day 4 and culture them to evaluate the antimicrobial performance *in vivo*. The results showed that the CTP + NIR group had few bacterial colonies, whereas the CT and CTP groups had significantly more bacterial colonies, and the control group showed severe bacterial infection (Figure 9G). Quantitative results showed that the bacterial survival rate in the CTP + NIR group was only 4.37%, which was significantly lower than that in the CTP group (49.73%) and the CT group (58.20%) (Figure 9H). The antimicrobial performance exhibited by the CTP + NIR group was attributed to the inherent antimicrobial capacity of the hydrogel and the photothermal-assisted antimicrobial effect 40, 66. Meanwhile, the CT_0.3_P hydrogel was also effective in forming a physical barrier against extra bacteria 67. Quantitative analysis of the wound healing rates showed that the CTP + NIR group had higher healing rates than the other groups at all time points. Especially on day 14, the healing rate was as high as 97.42%. In comparison, during the same period, the wound healing rates of the control, CT and CTP groups were 75.83%, 78.78% and 89.85%, respectively (Figure 9I). These results suggest that the CTP + NIR group had the function of promoting the wound repair process and increasing the healing rate, showing superior therapeutic effects than the other groups.

### Histological analysis

**Figure 10:**
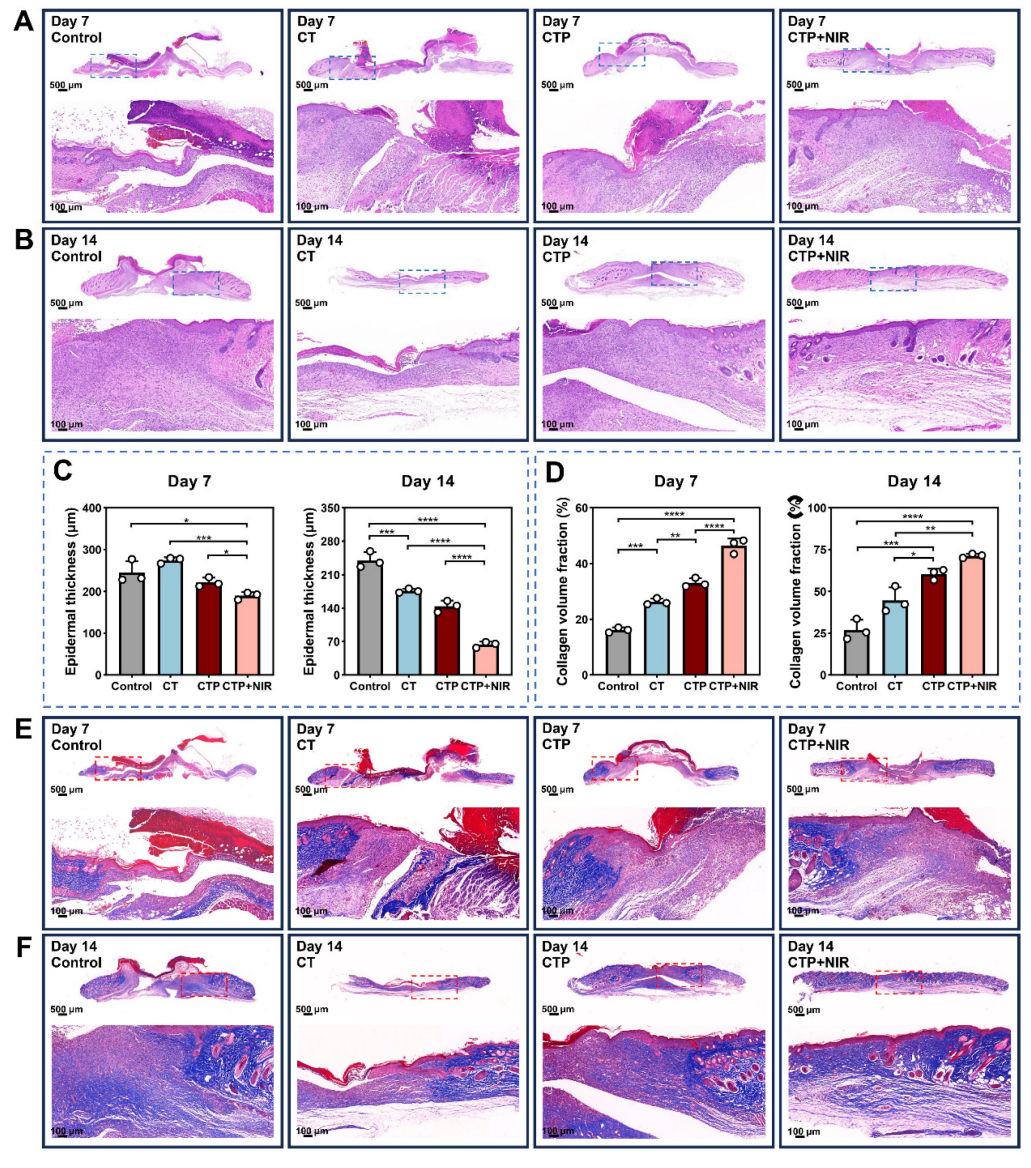
Histologic analysis. (A-B) H&E staining images on day 7 and day 14. (C) Statistical graphs of quantitative analysis of epidermal thickness. (D) Statistical graphs of quantitative analysis of collagen volume fraction (CVF). (E-F) Masson's trichrome staining images on day 7 and day 14 (*P < 0.05, **P < 0.01, ***P < 0.001, ****P < 0.0001, mean ± SD, n = 3).

Histological analysis of the wound area was performed by H&E staining and Masson trichrome staining on day 7 and day 14 after treatment 68. The results of H&E staining showed that inflammatory cell infiltration and fibroblast migration, which in turn contributed to the formation of granulation tissues, were observed in all groups on day 7 after treatment. The CTP + NIR hydrogel group presented the thickest granulation tissues. Meanwhile, the epidermal structures were significantly damaged in the control and CT groups, while the wound area in the CTP group showed a larger gap (Figure 10A). On day 14, the thickness of the granulation tissue increased in all groups. Particularly, the epidermis of the CTP + NIR group was completely regenerated, and the thickness of the dermis was consistent with that of the surrounding undamaged skin (Figure 10B). Quantitative results showed that both on day 7 and day 14, the CTP + NIR group differed significantly from the other groups in the thickness of the new epidermis. Especially on day 14 after treatment, the epidermal thickness of the CTP + NIR group was very close to that of the normal skin around the wound and much thinner than the other three groups (Figure 10C). In addition, considering that thickened epidermis is associated with the formation of adverse scars, the experimental results showed that CTP + NIR group not only accelerated wound healing by promoting epidermal reconstruction, but also effectively inhibited the formation of scars.

In the high glucose microenvironment of diabetic wound, the collagen deposition process is impaired, which severely hinders wound healing 69. Meanwhile, scar formation is closely related to ECM synthesis and remodeling in the dermis. The quantitative results of collagen volume ratio showed that the collagen deposition in the CTP + NIR group was significantly higher than the other three groups at both day 7 and day 14 (Figure 10D). It helped to promote the remodeling process of granulation tissue, which in turn effectively reduced the generation of scar tissue. Masson trichrome staining results showed that both on day 7 and day 14, the CTP + NIR group presented a more regular and orderly arrangement of collagen fibers. On day 14, the wound collagen fiber arrangement in the CTP + NIR group was almost the same as that of the surrounding normal skin, indicating that ECM and tissue remodeling were significantly improved. And the presence of blood vessels and hair follicles could be clearly observed in the regenerated skin tissue (Figure 10E-F). The above results suggest that CTP + NIR can structurally and functionally promote tissue regeneration and healing of diabetic infected wound.

### Analysis of CTP + NIR treatment for inhibiting scar formation in diabetic wound

**Figure 11:**
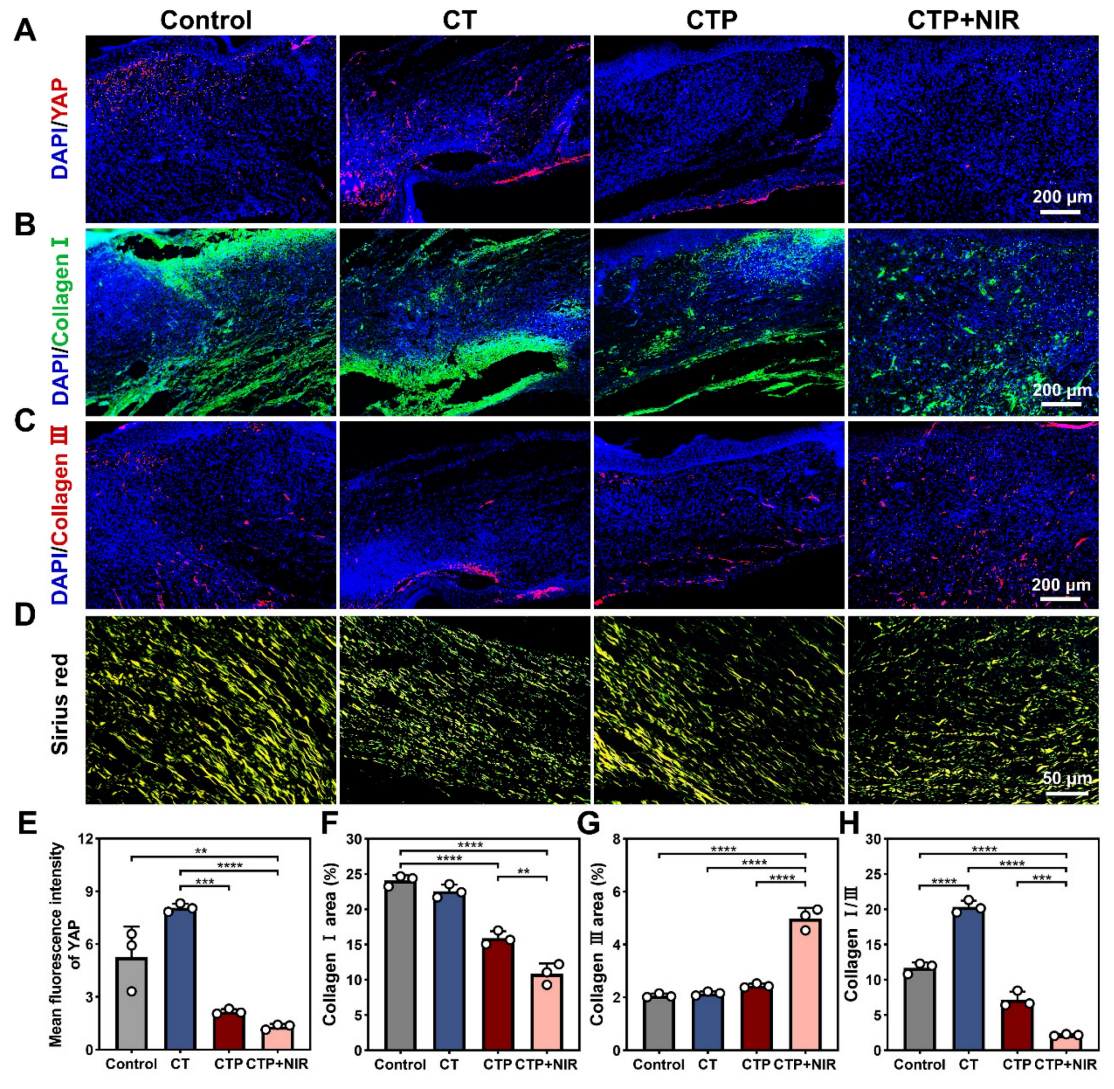
Effect of CTP + NIR treatment to inhibit scar formation in wound healing. Immunofluorescence staining images of A) YAP, B) collagen type I and C) collagen type III at the wound on day 14 after treatment. (D) Sirius red staining images, where collagen type I fibers were stained yellow and collagen type III fibers were stained green; Statistical graph of positive expression of E) YAP, F) collagen type I and G) collagen type III. (H) Statistical graph of the ratios of collagen I/III in different groups (**P < 0.01, ***P < 0.001, ****P < 0.0001, mean ± SD, n = 3).

Excessive formation of skin scars has become an urgent medical challenge. An ideal wound dressing should have the function of inhibiting scar formation while promoting wound healing 70. Fibroblasts play a central mediating role in the process of scar formation and are highly sensitive to mechanical signals. It was found that inhibition of YAP, a transcription factor sensitive to mechanical signals, effectively inhibited the activation of the transcription factor Engrailed-1 (En-1) during the wound healing process, thereby reducing the number of En-1-expressing fibroblasts, which in turn inhibited fibrosis and scar formation 71, 72. The expressions of YAP in the regenerated tissues of each group were detected by immunofluorescence staining. The results showed that compared with the control and CT groups, the intensity of red fluorescence was significantly reduced in the CTP and CTP + NIR groups (Figure 11A). Quantitative results showed that the CTP group significantly down-regulated YAP expression compared with the CT group. Notably, YAP expression was further down-regulated in the CTP + NIR group (Figure 11E), which was consistent with the H&E results. It suggests that CTP + NIR can inhibit the large amount of YAP expression at the wound, which in turn inhibits scarring.

Collagen is the main support structure of the skin, and changes in its composition and its ratio are usually the basis of proliferative scar formation. The content and ratio of collagen type I to collagen type III can indirectly reflect scar formation 73. The results showed that on day 14 after treatment, collagen type I deposition occurred in the control, CT, CTP and CTP + NIR groups, but the amount of collagen type I deposition varied among the groups. The amount of collagen type I deposition gradually decreased with the enhancement of ROS scavenging ability (Figure 11B). Quantitative analysis showed that collagen type I expression was significantly reduced in the CTP group and the CTP + NIR group, which was closely related to the excellent ROS scavenging property of CTP hydrogel. In the CTP + NIR group, the deposition of collagen type I was significantly lower than that in the other groups, and it was hypothesized that this phenomenon might be related to the creation of a more favorable microenvironment for wound healing by the CTP hydrogel with the assistance of NIR (Figure 11F). Meanwhile, the highest collagen type III deposition was observed in the CTP + NIR group (Figure 11C and Figure 11G). The ratios of collagen type I to collagen type III in the regenerated tissues of each group were further analyzed by Sirius red staining 74. Under polarized light, it was observed that the CTP + NIR group had the smallest area of collagen type I, while the area of collagen type III was more abundant (Figure 11D). Quantitative results showed that there was no significant difference between the ratio of collagen type I to collagen type III in control group (11.82) and the CT group (8.68). In contrast, the ratio in the CTP + NIR group was about 2.9 (Figure 11H), which was closest to normal skin 75. These results suggest that CTP + NIR treatment not only helps to promote re-epithelialization, but also effectively inhibits scar formation.

### Analysis of anti-inflammatory and angiogenic properties of CTP + NIR treatment in diabetic wound

**Figure 12:**
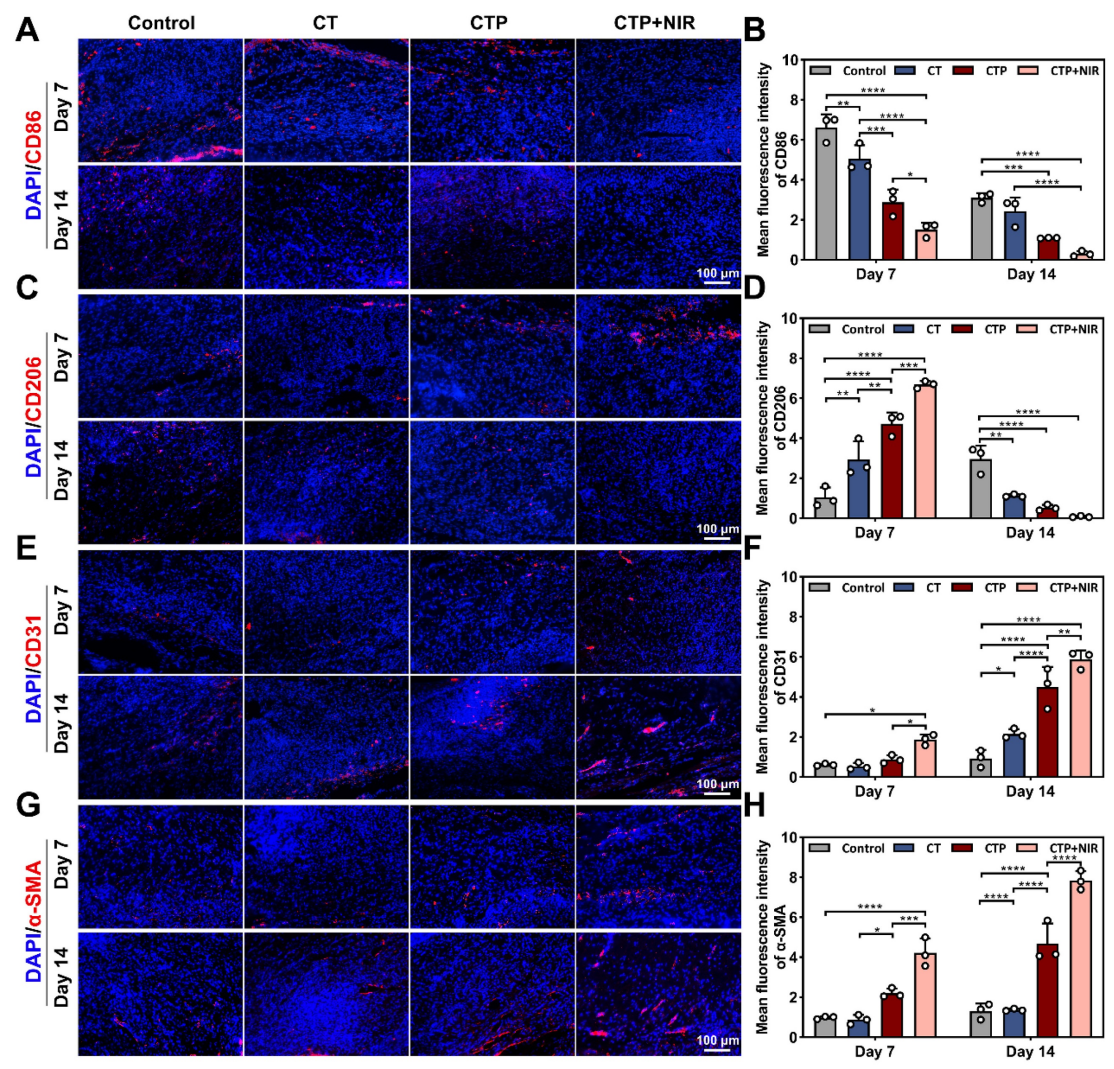
Anti-inflammatory and angiogenic effects of CTP + NIR treatment in wound healing. Immunofluorescence staining images of A) CD86, C) CD206, E) CD31 and G) α-SMA in regenerated tissues on day 7 and day 14 and statistical graphs of the mean fluorescence intensity of B) CD86, D) CD206, F) CD31, and H) α-SMA (*P < 0.05, **P < 0.01, ***P < 0.001, ****P < 0.0001, mean ± SD, n = 3),

During chronic diabetic wound healing, prolonged inflammatory response and immune dysfunction lead to impaired fibroblast function and angiogenesis 76. Macrophages play a key role in the inflammatory phase of wound healing, with a change in their phenotype from mainly pro-inflammatory (M1-type) to anti-inflammatory (M2-type) 77. On day 7 and day 14, the CD86 expression level was higher in the control group compared to the CT group, indicating that the wounds were still in the stage of a distinct inflammatory response. In contrast, the CD86 expression in the CTP group decreased significantly, and the level of inflammation was effectively controlled. Notably, the CD206 expression level was highest in the CTP + NIR group on day 7 and lowest on day 14 (Figure 12A-D). These results suggested that CTP hydrogel significantly decreased the proportion of M1-type macrophages while increasing the proportion of M2-type macrophages in the wound microenvironment. This not only helped to reduce the inflammatory response at the wound site, but also accelerated the wound healing process. Meanwhile, the combined application of CTP hydrogel and NIR can further enhanced its therapeutic effect.

Neovascularization can provide oxygen and nutrients to the wound area, which significantly contributes to chronic wound healing triggered by diabetes 78. CD31, a marker of vascular endothelial cells, plays a key role in angiogenesis during the proliferation and remodeling phases of wound healing 79. α-SMA (α-smooth muscle actin) is often used as a specific marker for vascular smooth muscle cells and is able to localize neovascular structures in regenerating tissues 38. The results showed that compared to the control, CT and CTP groups, the wounds in the CTP + NIR group showed more significant CD31 and α-SMA expression on day 7. In addition, on day 14, angiogenesis was significantly enhanced in the CTP and CTP + NIR groups, and the highest levels of CD31 and α-SMA expression were observed in the CTP + NIR group (Figure 12E-H). This indicated that CTP hydrogel effectively promoted angiogenesis on day 14, whereas CTP + NIR treatment had a stronger angiogenesis-promoting effect. In conclusion, by reducing the expression of inflammatory factors and simultaneously promoting the expression of CD31 and α-SMA, the CTP + NIR treatment significantly accelerated wound healing and had a more pronounced healing effect compared to commercial hydrogel.

### Mechanism analysis of CTP + NIR treatment to accelerate diabetic wound healing

**Figure 13:**
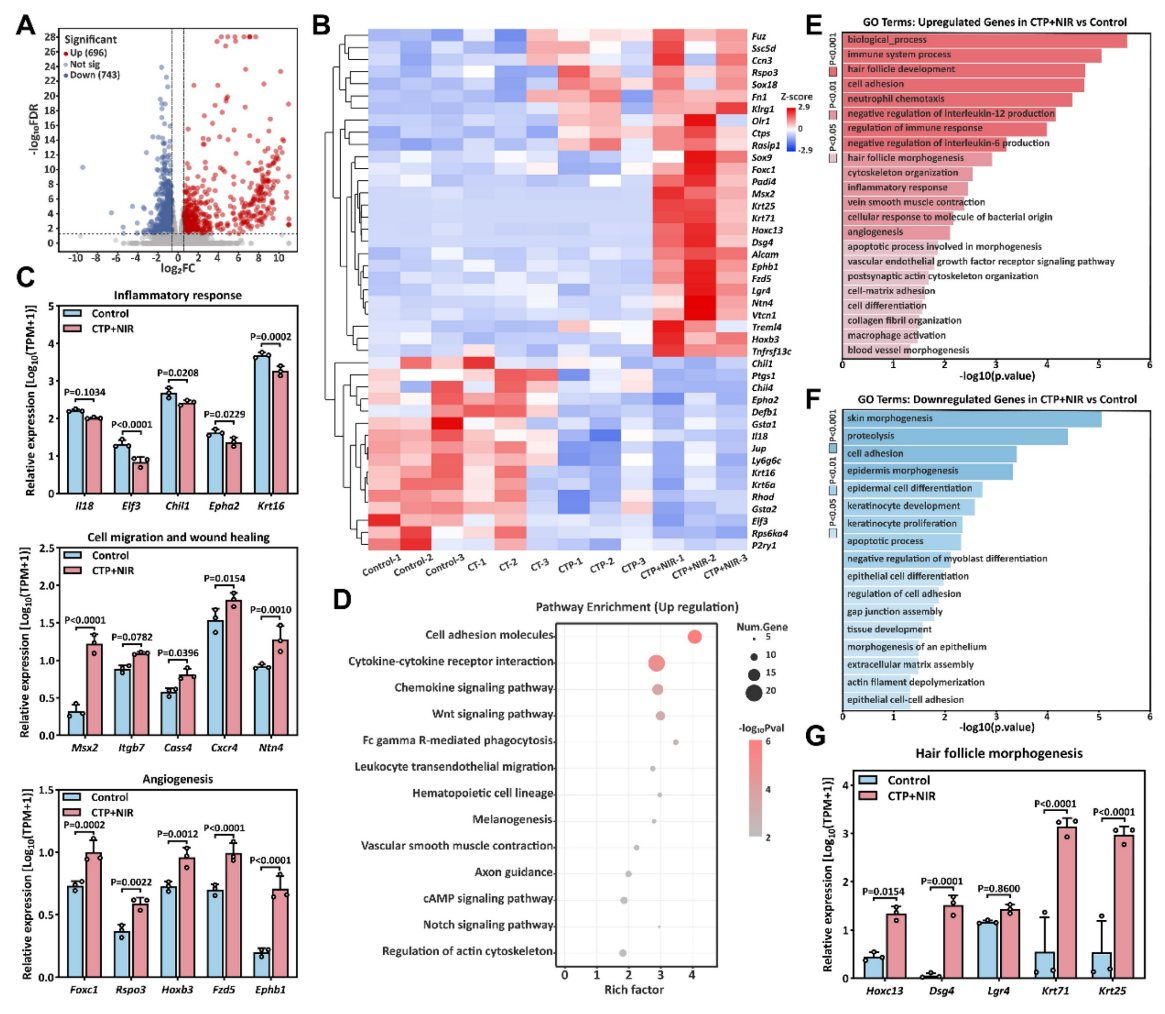
Mechanisms of CTP + NIR treatment to accelerate diabetes wound healing. (A) Volcano plot of differentially expressed genes in the control and CTP + NIR groups on day 7. (B) Heatmap of differentially expressed genes associated with wound healing. (C) Relative expression of genes associated with inflammation, cell migration and wound healing, and angiogenesis in the control and CTP + NIR groups on day 7. (D) KEGG pathway analysis of up-regulated genes in the control and CTP + NIR groups on day 7. (E) GO enrichment analysis of up-regulated genes in the control and CTP + NIR groups on day 7. (F) GO enrichment analysis of down-regulated genes in the control and CTP + NIR groups on day 7. (G) Relative expression of genes associated with follicle morphogenesis in the control and CTP + NIR groups on day 7 (mean ± SD, n = 3).

To further explore the mechanism of CTP + NIR treatment on diabetic wound healing, we collected wound regeneration tissues on day 7. Commercial hydrogel treatment of wound tissue was used as a control group for transcriptome sequencing analysis. Volcano plot revealed significant differences in gene expression levels between the two groups. Compared with the control group, 1439 differentially expressed genes (DEGs) were identified in the CTP + NIR treatment group, including 696 up-regulated genes and 743 down-regulated genes (Figure 13A). Further heat map showed that genes related to cell migration, angiogenesis and wound healing, such as Msx2, Ntn4, Foxc1, Rspo3 and Hoxb3, were significantly up-regulated in the CTP + NIR group. Meanwhile, genes associated with inflammation, such as Chil1, Epha2 and Krt16, showed significant down-regulation in the CTP + NIR group (Figure 13B-C). To further investigate the effects of CTP + NIR treatment on cell signaling mechanisms, we performed Kyoto Encyclopedia of Genes and Genomes (KEGG) enrichment analysis of differentially expressed genes. The results showed that the expression levels of signaling pathways such as Cell adhesion molecules, Wnt, cAMP, and Notch, which are closely related to anti-inflammation, cell migration, and wound healing, were significantly upregulated (Figure 13D). In addition, we performed Gene Ontology (GO) enrichment analysis of the differentially expressed genes. The results showed that genes associated with wound healing, including positively regulated genes for cell differentiation, tissue remodeling, angiogenesis, and follicle morphogenesis, exhibited upregulation in the CTP + NIR group (Figure 13E). In contrast, genes associated with fibrosis and related biological processes, such as extracellular matrix, intercellular junctions, and apoptotic processes, were all significantly down-regulated in this group (Figure 13F). This result suggests that CTP + NIR treatment has potential in inhibiting scar formation. Notably, it was also observed that genes associated with follicle morphogenesis, such as Dsg4, Krt71, and Krt25, were significantly up-regulated in the CTP + NIR group (Figure 13G). It was laterally verified that the CTP + NIR group was able to effectively inhibit scar formation, further supporting the therapeutic effect of the treatment in promoting diabetic wound healing.

Through transcriptome sequencing and analysis, we deeply explored the molecular mechanisms by which CTP + NIR treatment improves the diabetic wound microenvironment. Overall, the improvement of the wound microenvironment was mainly due to the collaborative effect of CTP hydrogel and NIR. On the one hand, both CTP hydrogel and NIR were able to promote angiogenesis, thus improving oxygen and nutrient supply to the wound area. On the other hand, CTP hydrogel provided a more favorable microenvironment for wound healing by performing antioxidant and anti-inflammatory effects and promoting cell migration. In summary, the CTP + NIR treatment not only accelerated the process of wound healing, but also played an important role in inhibiting scar formation and promoting tissue repair by regulating relevant signaling pathways and gene expressions.

## Conclusions

In summary, we developed a multifunctional hydrogel using a natural material, PHL, with a functional polymer through a simple mixing process and free of any extra cross-linking agents or functional modifications. This development approach integrates the unique bioactivities of PHL into a hydrogel system designed specifically for diabetic wound healing. The CTP hydrogel exhibited excellent swelling, adhesion, shape adaptability, antioxidant, coagulation property and biocompatibility. Especially important is its ability to promote cell migration and angiogenesis under conditions of high-glucose and oxidative stress conditions. Further results *in vivo* showed that CTP hydrogel had good hemostatic ability. In addition, the hydrogel combined with NIR treatment has efficient antibacterial effect. It also significantly promoted diabetic wound healing and tissue regeneration by reducing inflammation and promoting collagen deposition and angiogenesis. What's more, CTP hydrogel performed particularly well in reducing the ratio of collagen type I to collagen type III at the wound site, suggesting its potential to reduce scar formation during the healing process. This hydrogel provided a comprehensive and efficient solution for rapid healing of diabetic wounds with little or no scarring, offering a new way of thinking and approaching the field of diabetic wound treatment.

## Supplementary Material

Supplementary figures.
