# Supplementary material for: Naturally derived hydrogel with antioxidant, angiogenesis and photothermal effect to accelerate infected diabetic wound healing and reduce scar formation
Source: Theranostics. 2025 Sep 8;15(18):9838–61. doi: 10.7150/thno.117179 (PMC12486428; doi:10.7150/thno.117179)
Supplement: Supplementary file 1 — Supplementary figures. [file thnov15p9838s1.pdf]

Naturally derived hydrogel with antioxidant, angiogenesis and photothermal effect to accelerate infected diabetic wound healing and reduce scar formation

Mengyu Yang[1], Pengyuan Liu[1], Pei Cheng[1], Chenghao Li[1], Jingmei Liu[1], Haifeng Sun[2], Fangxia Guan[1], Minghao Yao[1]

[1]School of Life Science, Zhengzhou University, 100 Science Road, Zhengzhou 450001, P. R. China

[2]Department of Colorectal and Anal Surgery, The First Affiliated Hospital of Zhengzhou University, Zhengzhou, 450001, P. R. China

*E-mail: yao453343550@126.com, guanfangxia@126.com, sunhaifeng@zzu.edu.cn

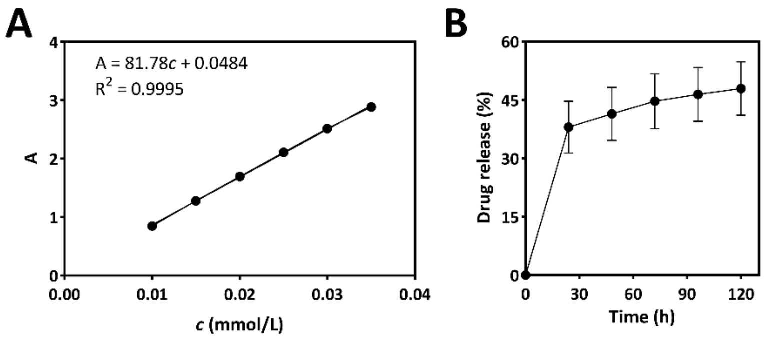

Figure S1. A: Standard curve of PHL; B: Release of PHL *in vitro* (Mean ± SD, n = 3)

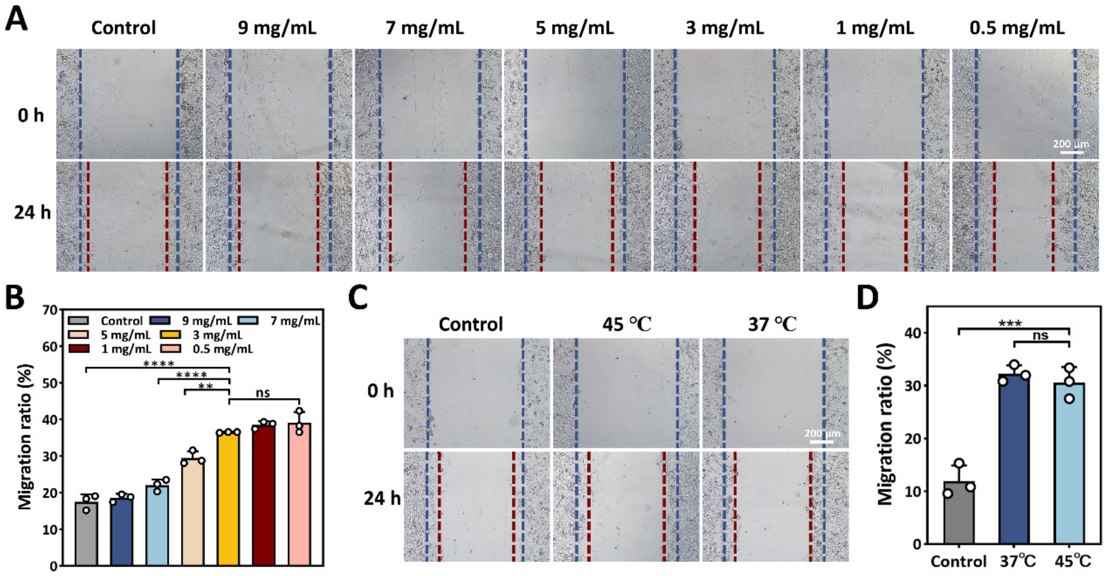

Figure S2. A: Images of cell migration promoted by different concentrations of PHL; B: Statistical graph of cell migration rate of different concentrations of PHL; C: Images of cell migration promoted by 3 mg/mL PHL after treatments at 45°C and 37°C, respectively; D:

Statistical graph of cell migration rate of 3 mg/mL PHL at different temperatures (**$P < 0.01$, ***$P < 0.001$, ****$P < 0.0001$, Mean ± SD, n = 3)

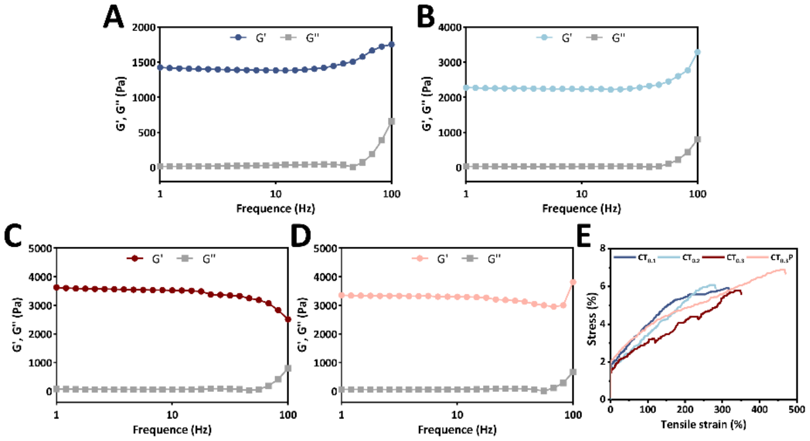

Figure S3. Rheological test results of A) $CT_{0.1}$, B) $CT_{0.2}$, C) $CT_{0.3}$, D) $CT_{0.3}$P hydrogels; E Tensile stress-strain test results of each group of hydrogels
